# Insights into the antimicrobial and antibiofilm activities of ionic liquids and their mechanisms of action

**DOI:** 10.1007/s00253-026-13728-x

**Published:** 2026-03-20

**Authors:** Hadeer M. Bedair, Israa A. Elmasry, Fotouh R. Mansour

**Affiliations:** 1 Department of Microbiology and Immunology, College of Pharmaceutical Sciences and Drug Manufacturing, Misr University for Science and Technology, the 6Th of October City, 12566 Egypt; 2 Pharmaceutical Analytical Chemistry Department, Faculty of Pharmacy, Tanta University, Tanta, 31111 Egypt; 3 Department of Medicinal Chemistry, Faculty of Pharmacy, King Salman International University (KSIU), South Sinai, Egypt

**Keywords:** Antibacterial resistance, Antifungal resistance, Antibiofilm, Ionic liquids, Mechanism of action

## Abstract

The need for novel antibacterial and antifungal treatments originates from the growing incidence of multidrug-resistant organisms, which is a serious public health concern. Conventional antibacterial and antifungal drugs are restricted by their narrow activity spectrum and lack of appropriate targets. The rise of antifungal drug resistance has made numerous commonly used drug classes ineffective, greatly complicating the clinical treatment of fungal infections. Additionally, bacteria and fungi that form biofilms are considered a major public health issue because they evade host defenses and contribute to antimicrobial resistance. To overcome these challenges, novel alternatives such as ionic liquids (ILs) have been developed. These compounds exhibit antifungal and antibacterial properties and can additionally function as antibiofilm agents. This review explores the nature of these materials, their methods of preparation, their uses, and their advantages. Furthermore, the antimicrobial activities of various ILs toward Gram-positive bacteria, Gram-negative one, and fungal species (spp) are discussed. Additionally, different mechanisms of action of these materials, along with their antibiofilm activity, will be discussed in this review.

**Supplementary Information:**

The online version contains supplementary material available at 10.1007/s00253-026-13728-x.

## Introduction

Antimicrobial resistance has become a major global health challenge, reducing the effectiveness of conventional antibiotics and complicating the treatment of bacterial and fungal infections (Nikfarjam et al. 2021; Di Martino 2022). The spread of resistance is driven by factors such as the misuse of antibiotics, horizontal gene transfer, and the persistence of resistant strains within the human microbiota (Report 2014; Ahmed et al. 2024). Similarly, fungal pathogens are increasingly resistant to conventional antifungal therapies, further complicating clinical management, especially in immunocompromised patients (Peyclit et al. 2021; Martins-Santana et al. 2023). These facts highlight the urgent need for novel antimicrobial agents capable of overcoming both bacterial and fungal resistance.

Ionic liquids (ILs) are examples of these new materials that have emerged as promising multifunctional materials with potent antimicrobial properties (Bedair et al. 2025). ILs are liquid salts below 100 °C, composed of a bulky organic cation and an inorganic or organic anion whose combinations can be “designed” to tune properties and biological activity (Flieger et al. 2020; Singh and Savoy 2020). Based on their ionic constituents, ILs are broadly classified into cationic, anionic, and zwitterionic ILs, with cationic ILs being the most extensively studied for antimicrobial applications (Flieger et al. 2020; García et al. 2023). Common cations include imidazolium, pyridinium, ammonium, phosphonium, and cholinium, while frequently used anions include halides (Cl⁻, Br⁻), tetrafluoroborate (BF₄⁻), hexafluorophosphate (PF₆⁻), and organic carboxylates (Gonçalves et al. 2021; Vereshchagin et al. 2021; García et al. 2023). Among these, imidazolium- and ammonium-based ILs have demonstrated notable antibacterial and antibiofilm activities, primarily due to their ability to disrupt bacterial cell membranes and interfere with biofilm matrix integrity.

Although salts are generally solids because of the strong electrostatic attraction between positively charged cations and negatively charged anions, this is not the case for this interesting class of compounds. In ILs, the irregular shapes of the ions and their poor packing efficiency weaken these electrostatic forces, preventing the formation of a stable solid lattice. At the same time, this structural disorder makes ILs distinct from conventional liquids, imparting unusually high viscosities, negligible vapor pressure, high thermal and chemical stability, and wide electrochemical windows. ILs are therefore considered advantageous and environmentally friendly solvents that offer an alternative to commonly used volatile organic solvents (Abdallah et al. 2022; Abdelaziz et al. 2023).

Beyond their chemical and industrial applications, ILs have attracted attention from biochemists, microbiologists, and medical researchers due to their antimicrobial and antibiofilm properties (Nikfarjam et al. 2021; Bedair et al. 2024a). The structural versatility of ILs allows them to interact with bacteria and fungi, providing novel opportunities to address infections caused by antibiotic- and antifungal-resistant pathogens. The antimicrobial effectiveness of ILs arises from multiple interconnected mechanisms. Initially, ILs adsorb onto bacterial cell surfaces due to their strong affinity for membrane components. Subsequently, electrostatic interactions occur, leading to the inactivation of membrane-associated proteins and interactions with phospholipid head groups. ILs are also capable of penetrating and destabilizing the phospholipid bilayer, resulting in membrane disruption and leakage of intracellular contents. Ultimately, these combined effects culminate in cell wall disintegration and bacterial cell lysis (Nikfarjam et al. 2021; Bedair et al. 2024a). The antimicrobial efficacy of ILs is strongly influenced by the nature of both the cation and anion, as well as alkyl chain length, which allows their physicochemical and biological properties to be finely tuned for targeted antibacterial and antibiofilm application (Vereshchagin et al. 2021; García et al. 2023).

ILs can be synthesized using conventional methods at room temperature or non-conventional approaches, including microwave (MW) irradiation and ultrasonic-assisted (US) techniques (Ratti 2014; Singh and Savoy 2020). Among these techniques, MW irradiation offers rapid heating, high atom efficiency, enhanced product selectivity, and short reaction times without solvents. US synthesis accelerates reactions at the interface of immiscible liquids, improves energy efficiency, achieves high product yields, and avoids solvent use. These methods support eco-friendly and sustainable production of ILs, aligning with green chemistry principles (Singh and Savoy 2020).

This review provides a comprehensive overview of the antibacterial and antifungal properties of ILs, detailing their synthesis methods, key advantages, and representative examples of their antimicrobial effects against a range of microorganisms, including Gram-positive and Gram-negative bacteria as well as diverse fungal spp. Furthermore, it will elaborate on the distinct mechanisms of action exhibited by different types of ILs.

**Fig. 1:**
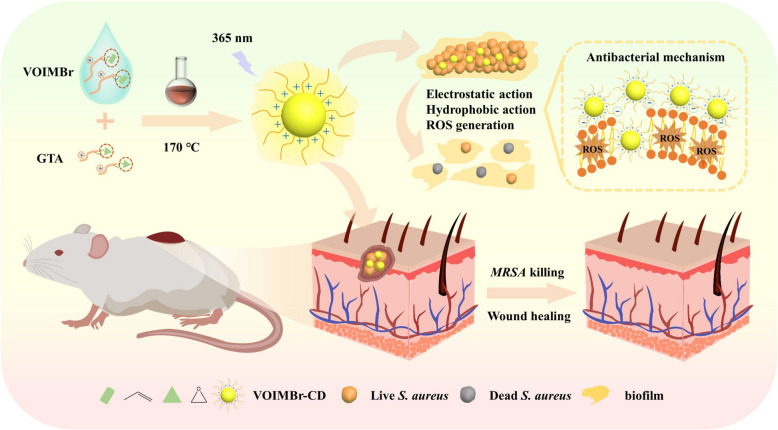
The synthesis procedure of VOIMBr-CD and its subsequent application in promoting wound healing and preventing biofilm formation. Reproduced with permission from Gao et al. (2024a)

Several studies reported the use of ILs against Gram-positive bacteria (Brunel et al. 2018; Rita Pereira et al. 2022; Cagide et al. 2024; Novello et al. 2024; Rackov et al. 2024; Wojcieszak et al. 2024; Zhou et al. 2024). A variety of ILs have been evaluated against Gram-positive cocci, particularly *Staphylococcus aureus* (*S. aureus*), a pathogen associated with numerous inflammatory conditions (Bedair et al. 2024b). Additionally, methicillin-resistant *S. aureus* (MRSA) and *Enterococcus* spp. were also used as models for Gram-positive bacteria. For Gram-positive rods, *Bacillus subtilis* (*B. subtilis*) and *B. cereus* were used. While certain ILs exhibited low antimicrobial activity, others were even more efficient than conventional antibiotics. Gao and his coworkers (2024a) synthesized IL-derived carbon dots (IL-CDs) through vinyl and epoxy group reactions, resulting in improved antimicrobial efficacy. It was observed that the vinyl imidazole, bromooctane-carbon dot (VOIMBr-CD) retained the antibacterial activity of these IL-CDs, which was attributed to the positively charged imidazolium group and long alkyl chain. Furthermore, VOIMBr-CD exhibited selective antibacterial activity against Gram-positive bacteria such as *S. aureus* more than the Gram-negative *Escherichia coli* (*E. coli*). This was attributed to the loose anionic cell wall of the Gram-positive bacteria that facilitated quicker binding and penetration, while the lipopolysaccharide dense membrane in Gram-negative bacteria prevented VOIMBr-CD from diffusing inward. Interestingly, the antibacterial efficacy of VOIMBr-CD against MRSA exceeded vancomycin antibiotic (Van); they proved that by comparing the antibacterial activity of the same concentration (20 μg/mL) of both VOIMBr-CD and Van using the plate counting method, after 2 h incubation period with MRSA, (VOIMBr-CD)-treated MRSA was completely eradicated, while Van-treated cells had a survival rate of 31.48%. Similar results were observed in mouse models with MRSA-infected wounds; after 12 days of treatment, the VOIMBr-CD treated group had a 98.89% wound healing rate, while the Van-treated group was 92.98%. This superior efficacy originated from the VOIMBr-CD mode of action that was dependent on the electrostatic interactions, hydrophobic interactions, and generation of reactive oxygen species (ROS) within bacterial cells, as shown in Fig. 1.

Wojcieszak and his coworkers tested a series of surface-active ILs (SAILs) against a panel of different pathogens. The results revealed a promising antibacterial activity against the highly pathogenic bacteria *Enterococcus faecalis* (*E*. *faecalis)* ATCC 29212. It was noted that the dicationic compounds showed much higher activity against tested microorganisms in comparison with monocationic ones (Wojcieszak et al. 2024). The synergistic effect of ILs was shown in the study of Ferraz and his coworkers. ILs and organic salts (OSILs), which contained anionic penicillin G [*seco*-Pen] and amoxicillin [*seco*-Amx] hydrolysate derivatives, were synthesized (Ferraz et al. 2020). The results showed a promising and beneficial effect on the drug delivery of the modified active pharmaceutical ingredients when combined with hydrophobic organic cations. In this regard, the antimicrobial resistance to the standard β-lactam antibiotics was drastically reduced in vitro. The synergistic interaction of OSILs with β-lactam antibiotics was quantified using the relative decrease of inhibitory concentration**,** which was calculated by comparing the minimum inhibitory concentration (MIC) of the OSIL to that of the parent β-lactam (or its sodium salt) (Ferraz et al. 2020). It was revealed that OSILs could substantially enhance the activity of conventional β-lactams, reducing MIC values by 100 to 1000-folds, depending on the bacterial strain and the OSIL used (Ferraz et al. 2020). Table 1 summarizes the antibacterial activity of different ILs and IL-based materials against Gram-positive bacteria.

**Table 1:** Antibacterial activity of different ILs and IL-based materials against Gram-positive bacteria

Tested organism	Ionic liquids/ILs-based	Method of preparation	Antibacterial effects	Reference
*B. cereus*	Choline-based ionic liquids	NR*	MBC: > 10,000 mg/L	(Rita Pereira et al. 2022)
*B. cereus*	1,4phenylenebis(methanylylidene))bis(azanylydiene)bis(propane-3,1-diyl)bis(3- decyl-1H-imidazol-3-ium) bis bromide (3 g)	Solvothermal/Schiff base	MIC: 4.9 µg/mL	(ünver et al. 2024)
*B. subtilis*	1,4phenylenebis(methanylylidene))bis(azanylydiene)bis(propane-3,1-diyl)bis(3- decyl-1H-imidazol-3-ium) bis bromide (3 g)	Solvothermal/Schiff base	MIC: 4.9 µg/mL	(ünver et al. 2024)
*B. subtilis* ATCC 6633	Poly([C_12_VIm][Br])	Solvothermal	MIC:0.022 mg/mL	(Rackov et al. 2024)
*E. faecalis*	1,4phenylenebis(methanylylidene))bis(azanylydiene)bis(propane-3,1-diyl)bis(3- decyl-1H-imidazol-3-ium) bis bromide (3 g)	Solvothermal/Schiff base	MIC: 2.4 µg/mL	(ünver et al. 2024)
*E. faecalis* ATCC 29212	[DC_4_][dicamba]_2_	Metathesis reaction	MIC:0.003 mmol/L	(Wojcieszak et al. 2024)
*E. aerogenes* ATCC 13,048	TPA-P derivatives	Microwave-assisted	MIC: 16 mg/L	(Brunel et al. 2018)
*E. faecium* 20,477	TPA-P derivatives	Microwave-assisted	MIC: 1 mg/L	(Brunel et al. 2018)
MRSA	VOIMBr-CD	Hydrothermal and stirring	Plate counting: at 20 μg/mL, completely eradicated MRSA	(Gao et al. 2024a)
MRSA ATCC 43300	Dodecyltriphenyl phosphonium bromide	Quaternization	MIC: less than/equal 0.25 µg/mL	(Cagide et al. 2024)
MRSA ATCC 43300	[C_2_OHMIM][seco-Amx]	Stirring/ion-exchange	Broth micro dilution: MIC 5 mM	(Ferraz et al. 2020)
*Micrococcus luteus* JR-10	[Tbp][Dodec]	Stirring at room temperature	MIC: 0.60 mmol/L MBC: 0.60 mmol/L	(Panić et al. 2024)
*L. monocytogenes*	C_14_mimBF_4_	The alkylation of the MIM ring and metathesis reactions	MIC: 4.58 μg/mL MBC: 4.58 μg/mL	(Novello et al. 2024)
*S. aureus*	[TTP]_2_[Fl]	Metathesis reaction	MBC: 46 ± 7.6 µmol/L	(Das et al. 2021)
*S. aureus*	1,4phenylenebis(methanylylidene))bis(azanylydiene)bis(propane-3,1-diyl)bis(3- decyl-1H-imidazol-3-ium) bis bromide (3 g)	Solvothermal/Schiff base	MIC: 4.9 µg/mL	(ünver et al. 2024)
*S. aureus* ATCC 25213	[DC4][dicamba]_2_	Metathesis reaction	MIC:0.006 mmol/L	(Wojcieszak et al. 2024)
*S. aureus* ATCC 2592	PBIC	Hydrothermal	MIC:32 µg/mL	(Zhou et al. 2024)
*S. aureus* ATCC 25923	Films of (P(VDF-TrFE) incorporated with [Emim][HSO_4_]	Solvent casting	Contact-killing assays: 99% bacterial elimination	(Carvalho et al. 2024)
*S. aureus* ATCC 25923	[Tbp][Dodec]	Stirring at room temperature	MIC: 0.60 mmol/L MBC: 1.20 mmol/L	(Panić et al. 2024)
*S. aureus* ATCC 25923	Poly([C_12_VIm][Br])	Solvothermal	MIC:0.09 mg/mL	(Rackov et al. 2024)
*S. aureus* ATCC 25923	PVA/CA/IL fibers	Solvothermal	Plate count: reduction in cell viability 50.73%	(P Libel et al. 2024)
*S. aureus* ATCC 25923	[C_2_OHMIM][seco-Amx]	Stirring/ion-exchange	MIC 0.05 mmol/L	(Ferraz et al. 2020)
*S. aureus* ATCC 29737	Thiazolium ILS RTLMCs	Deproteination, demineralization and deacetylation	AWD: 37.9 mm CFU: reduce the bacterial populations by up to 97.9% MIC: 1.20 μg/mL	(Binjawhar et al. 2024)
*S. aureus* ATCC6538	CABILs: [Ch][Lys] [Ch][Arg] [Ch][His]	Heating and stirring	Bacteriostatic rate: 81.198%	(Gao et al. 2024b)
*S. aureus* CIP 7625	TPA-P derivatives	Microwave-assisted	MIC: 1 mg/L	(Brunel et al. 2018)
*S. aureus* CMCC 26003	PVA/C_12_MPBr hydrogel	Stirring	Inhibition zone:17.15 mm	(Yu et al. 2020)
*S. aureus* MCCCB 26003	[VBIm][Br]	Solvothermal	Colony assay: no bacterial growths with PMAV3	(Wang et al. 2020)
*S. aureus* MTCC 87	C_10_mimCl	Synergistic interaction	MIC: 15620 µmol/L	(Siddiquee et al. 2021)
*S. aureus* SA1199	TPA-P derivatives	Microwave-assisted	MIC: 0.5 mg/L	(Brunel et al. 2018)
*S. aureus* SA1199B	TPA-P derivatives	Microwave-assisted	MIC: 0.5 mg/L	(Brunel et al. 2018)
*S. epidermidis* ATCC14990T	[BMIM][PF_6_]	The emulsion solvent diffusion	Fluorescence live/dead viability assay: 30% viable bacterial cells	(Takahashi et al. 2019)
*S. epidermidis* JR-07	[Tbp][Dodec]	Stirring at room temperature	MIC: 0.60 mmol/L MBC: 1.20 mmol/L	(Panić et al. 2024)
*S. epidermidis* NCIMB 8853 CECT 231	Cu@PIL1.2-h	Anion metathesis	98% inhibit bacterial growth	(Miralles-Comins et al. 2024)

*NR*, not reported; *VOIMBr-CD*, 1-vinylimidazole, bromooctane-carbon dot; *AWD*, agar well-diffusion; *[DC4][dicamba]*_*2*_, dicationic 3,6-dichloro-2-methoxy)benzoic acid; *MIC*, minimum inhibitory concentration; *MBC*, minimum bactericidal concentration; *TPA-P*, triphenylamine phosphonium; *Cu@PIL1.2-h*, heated copper-based nanoparticles within the polymeric ionic liquids; *[Tbp][Dodec]*, tetrabutylphosphonium dodecanoate; *CAGE*, choline and geranate; *bromide, [C*_*12*_*VIm][Br]*, 1-alkyl-3-vinylimidazolium bromide; *(P(VDF-TrFE)*, films of poly(vinylidene fluoride-trifluoroethylene); *[Emim][HSO*_*4*_*]*, 1-ethyl-3- methyl imidazolium hydrogen sulfate; *RTLMCs*, ruthenium-based low molecular weight chitosans; *C₁₄mimBF*₄, 1-tetradecyl-3-methylimidazolium tetrafluoroborate; PBIC, poly(butyl imidazole hexanoate); PVA, poly(vinyl alcohol); CA, citric acid; CABILs, choline-based amino acid ionic liquids; *[Ch][Lys]*, choline lysinate; *[Ch][Arg]*, choline argininate; *[Ch][His]*, choline histidinate; *[C*_*2*_*OHMIM][seco-Amx]*, 1-(2-hydroxyethyl)−3-methylimidazolium seco-amoxicillinate; *seco*, indicates ring-opened (β-lactam–opened) amoxicillin anion; *C12MPBr*,1-dodecyl-3-methylpyridinium bromide; *C*_*10*_*mimCl*, 1-decyl-3-methylimidazolium chloride; *[TTP]*_*2*_*[Fl]*, trihexyltetradecyl phosphonium coupled with dianionic fluorescein; *[BMIM][PF*_*6*_*]*, 1-butyl-3-methylimidazolium hexafluorophosphate; *PMAV3*, poly(MMA-co-AM-co-[VBIm]Br); *MMA*, methyl methacrylate; *AM*, acrylamide**;***[VBIm]Br*, 1-vinyl-3-butylimidazolium bromide

## Mechanism of action of ILs

**Fig. 2:**
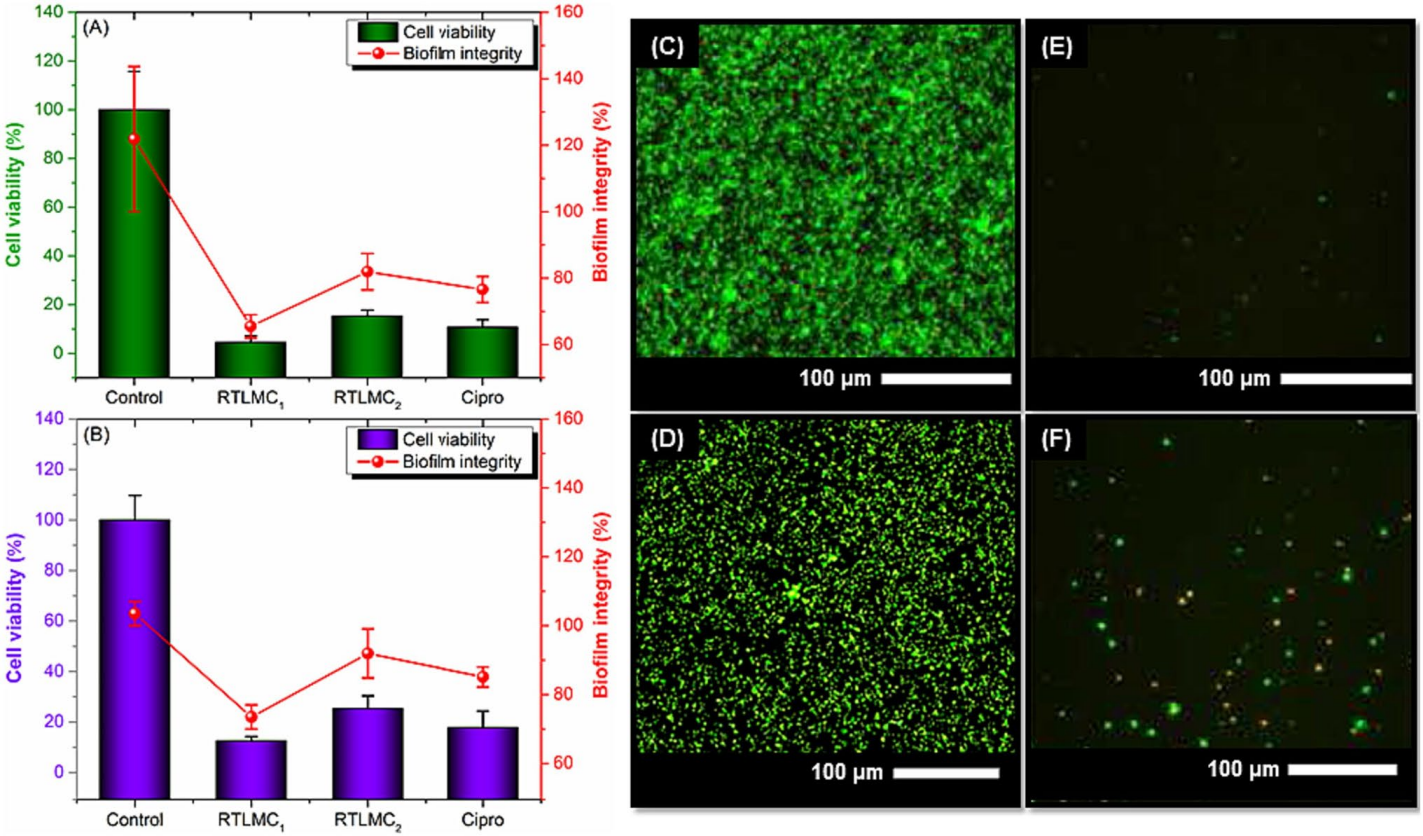
The impact of ruthenium thiazolium low molecular weight chitosans (RTLMCs) on pre-formed biofilms of *S. aureus* (**B**, **D**, and **F**) and *E. coli* (**A**, **C**, **E**) was evaluated. After allowing biofilms to develop on polystyrene surfaces for 24 h, nanocomposites (25 µg/mL) were introduced for treatment. Biofilm degradation was assessed using crystal violet staining, while cell viability and metabolic activity were determined via resazurin staining (**A**, **B**). The x-axis represents treatment groups: the untreated biofilm (negative control), chloride-based RTLMC1, hexafluorophosphate-based RTLMC2, and ciprofloxacin (positive control). Data represents the mean ± standard error from two independent experiments. Ciprofloxacin served as the positive control. Reproduced with permission from Binjawhar et al. (2024)

From a chemical perspective, ILs have a charged hydrophilic head group and a hydrophobic tail. They tend to self-assemble and form aggregates, which makes them amphiphilic in nature and exhibit aggregation behavior similar to conventional surfactants (Rackov et al. 2024). This explains why ILs destabilize and disintegrate the phospholipid bilayer in biological membranes. Consequently, increasing the alkyl chain length raises lipophilicity, which then affects the bacterial cytoplasmic membrane by causing changes in the structural and dynamic properties of the outer layers, ultimately resulting in disruption and loss of membrane integrity (Rita Pereira et al. 2022; Rackov et al. 2024). Additionally, the kind of anion in the ILs also has a role in controlling bacterial growth (Novello et al. 2024). Moreover, the effectiveness of the antibacterial properties of ILs is different according to the structural difference between Gram-negative and Gram-positive bacteria. It was reported that, usually, different ILs are considered more successful in combating Gram-positive bacteria. This could be explained by the fact that in Gram-positive bacteria, their structure is characterized by a substantially thicker, porous cell wall composed of peptidoglycan layers connected by a negatively charged teichoic acid, which gives the cell wall its porosity. On the other hand, in Gram-negative bacteria, the cell wall is composed of two layers: a relatively thin inner layer composed of peptidoglycan and an outer layer composed of lipopolysaccharides that are negatively charged. As a result, it is thought that the hydrophobic portion of IL has a higher chance of entering the porous peptidoglycan, rupturing the cell membrane, and resulting in cell death, while Gram-negative bacteria are shielded from the entry of ILs by their low permeability in their outer membrane (Novello et al. 2024). Another mechanism of action of the ILs is attributed to having the ability to alter the biochemical gradients that exist between the cytoplasm of the bacterial cell and the outside world, which can cause extracellular materials to enter the cytoplasm or intracellular contents to diffuse out of the cell (Nikfarjam et al. 2021). Furthermore, the antibacterial mechanism of various ILs is due to the electrostatic interaction between the cationic moieties in ILs and the bacterial cell wall’s phosphate groups. It is believed that these chemicals’ hydrophobic regions break bacteria’s lipid membranes, disrupting them and causing cell death (Zheng et al. 2016). According to the components of ILs, it was observed that imidazole-based ILs have demonstrated synergistic antimicrobial activity against both Gram-positive and Gram-negative bacteria when combined with small-molecule antibiotics (Wu et al. 2023). Certain imidazolium-based ILs have demonstrated notable antibacterial activity, which is largely attributed to their amphiphilic nature (Busetti et al. 2010). Moreover, imidazolium-based ILs have shown strong efficacy in inhibiting the growth of pathogenic, non-pathogenic, and drug-resistant strains of both bacteria and fungi (Cornellas et al. 2011; Florio et al. 2019; Garcia et al. 2020). In conclusion, ILs have different ways to work against pathogens, such as disrupting bacterial cell membranes, interacting with cellular components, and causing oxidative stress. Figure 2 below summarizes the different mechanisms of action of ILs.

### Antimicrobial in vitro activity of ILs

#### Antibacterial activity against Gram-negative bacteria

**Fig. 3:**
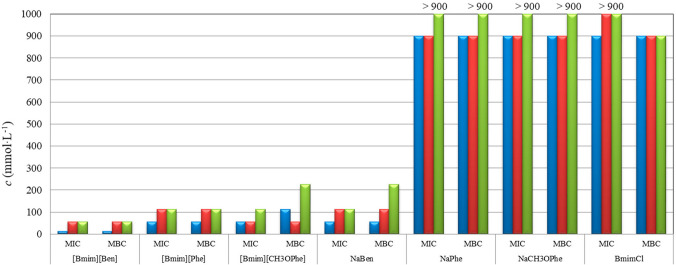
Antifungal effects of the evaluated ILs: [Bmim][Ben], 1-butyl-3-methylimidazolium benzoate; [Bmim][Phe], 1-butyl-3-methylimidazolium phenylacetate; [Bmim][CH3OPhe], 1-butyl-3-methylimidazolium 4-methoxyphenylacetate; their corresponding sodium salts: NaBen, sodium benzoate; NaPhe, sodium phenylacetate; NaCH3OPhe, sodium 4-methoxyphenylacetate; and the IL-precursor BmimCl, 1-butyl-3-methylimidazolium chloride against *P. verrucosum* (blue), *A. flavus* (red), and *A. parasiticus* (green). The corresponding sodium salts and the IL-precursor BmimCl were used as comparative standards. The x-axis represents the evaluated ILs and the biological parameters used to assess their antifungal activity: minimum inhibitory concentration (MIC) and minimum bactericidal concentration (MBC), while the y-axis represents the corresponding concentration in mmol/L. Reproduced with permission from Cako Bagany et al. (2024) under Creative Commons terms

Gram-negative bacteria are a major clinical concern due to their increasing resistance to commonly used antibiotics, which limits treatment options and complicates infection management (Rangarajan and Venkataraman 2020). Multidrug-resistant strains of *E. coli*, *Klebsiella pneumoniae* (*K. pneumoniae*), *Pseudomonas aeruginosa (P. aeruginosa)*, *Acinetobacter baumannii (A. baumannii)*, *Salmonella enterica* serovar Typhimurium, and *Proteus mirabilis* (*P. mirabilis*) are particularly challenging, as they can survive conventional therapies and rapidly acquire or transmit resistance determinants (Vuotto et al. 2014; Campos et al. 2016; Rangarajan and Venkataraman 2020). ILs have demonstrated antibacterial and antibiofilm activity against Gram-negative pathogens, including multidrug-resistant strains, by targeting cell membranes, disrupting biofilms, and enhancing the efficacy of existing antibiotics (Nikfarjam et al. 2021; Bedair et al. 2024a). A panel of Gram-negative bacteria was subjected to tests using several ILs and IL-based materials. According to the literature, one of the most promising ILs towards Gram-negative bacteria was reported in the study of Novello and his coworkers (Novello et al. 2024). Methylimidazolium ILs that incorporated long alkyl chain in their cations were developed with tetrafluoroborate (BF_4_) and the 1, 3-dimethyl-5-sulfoisophthalate as counter ions. These synthesized ILs were blended with poly vinyl chloride (PVC) to make PVC/IL film; this process involved solvent casting where the pellet of the PVC was dissolved in tetrahydrofuran and ILs were added in different weight percentages (0.5, 1.0, 5wt%). After stirring and evaporation, the formed films were used as self-disinfectant PVC materials to produce medical and surgical supplies such as syringes, catheters, and prosthetic limbs. The antimicrobial, anticancer, and cytotoxic activities of these ILs were investigated. The in vitro results showed that the antimicrobial activities of those ILs against *E. coli* and *Pseudomonas fluorescens* (*P. fluorescens)* strains were higher in the series that contained the BF_4_ anion and increased with the increase in the methylimidazolium cation alkyl chain length. However, the elongation of the alkyl chain beyond C16 induced a decrease in antimicrobial activity, indicating a cut-off effect. The results provided a strong support for the potential utilization of ILs in biomedical applications, especially as antibacterial agents to resolve the challenge of antibiotic resistance (Novello et al. 2024). In the study of Binjawhar and his coworkers (Binjawhar et al. 2024), they developed a novel thiazolium IL-functionalized low molecular weight chitosans (TLMCs). These TLMCs were used as reducing and encapsulating agents to create biofunctionalized TLMC-based ruthenium (RTLMCs) nanocomposites. These materials were tested in vitro for antimicrobial and anti-biofilm activities against *E. coli* as a model for Gram-negative bacteria. The results showed that the ability of these nanocomposites to synergistically inhibit bacterial growth and inhibit biofilm progression was substantial, as illustrated in Fig. 3.

The MIC value of RTLMC against *E. coli* was 0.68 μg/mL. Scanning electron microscope analysis indicated that RTLMCs’ greater activity was due to their capacity to cause damage to the bacterial outer membrane, which permitted internal contents to leak out and ultimately resulted in the organism’s death. The antibiofilm tests demonstrated that RTLMCs killed cells in submerged cultures or in preexisting biofilms in addition to preventing bacterial cells from adhering to coated surfaces and forming biofilms. These results suggested that RTLMCs may be employed to prevent and eradicate bacterial biofilms (Brunel et al. 2018; Rita Pereira et al. 2022; Cagide et al. 2024; P Libel et al. 2024; Wojcieszak et al. 2024). Table 2 shows more detailed information related to the antibacterial activities of different ILs and ILs-based materials towards Gram-negative bacteria.

**Table 2:** Antibacterial activity of different ILs and ILs-based materials against Gram-negative bacteria

Tested organism	ILs	Method of preparation	Antibacterial effects	Reference
*A. baumannii* ATCC 19,606	TPA-P derivatives	Microwave-assisted	MIC: 16 mg/L	(Brunel et al. 2018)
*A. baumannii* ATCC 19606	Dodecyltriphenyl phosphonium bromide	Quaternization	MIC:2 µg/mL	(Cagide et al. 2024)
*E. coli*	C14mimBF_4_	The alkylation of the MIM ring and metathesis reactions	MIC: 4.58 μg/mL MBC: 9.16 μg/mL	(Novello et al. 2024)
*E. coli*	[TTP][HFl]	Metathesis reaction	MBC: 85 ± 8.2 µmol/L	(Das et al. 2021)
*E. coli*	1,4phenylenebis(methanylylidene))bis(azanylydiene)bis(propane-3,1-diyl)bis(3- decyl-1H-imidazol-3-ium) bis bromide (3 g)	Solvothermal/Schiff base	MIC: 4.9 µg/mL	(ünver et al. 2024)
*E. coli ATCC* 25922	[VBIm][Br]	Solvothermal	Colony assay: no bacterial growths with PMAV3	(Wang et al. 2020)
*E. coli CGMCC* 1.12883	PVA/C_12_MPBr hydrogel	Stirring	Inhibition zone: 16.02 mm	(Yu et al. 2020)
*E. coli* ATCC 10536	Thiazolium ILs RTLMCs	Deproteination, demineralization and deacetylation	AWD:46.8 mm MIC:0.68 μg/mL CFU: reduce the bacterial populations by up to 99.4%	(Binjawhar et al. 2024)
*E. coli* ATCC 25922	Poly([C_12_VIm][Br])	Solvothermal	MIC:0.09 mg/mL	(Rackov et al. 2024)
*E. coli* ATCC 25922	Dodecyltriphenyl phosphonium bromide	Quaternization	MIC: less than/equal 0.25 µg/mL	(Cagide et al. 2024)
*E. coli* ATCC 25922	[DC4][dicamba]_2_	Metathesis reaction	MIC:0.005 mmol/L	(Wojcieszak et al. 2024)
*E. coli* ATCC 25922	[C_2_OHMIM][seco-Amx]	Stirring/ion-exchange	MIC: 5 mmol/L	(Ferraz et al. 2020)
*E. coli* ATCC 8739	Films of (P(VDF-TrFE) incorporated with [Emim][HSO_4_]	Solvent casting	Contact-killing assays: 99% bacterial elimination	(Carvalho et al. 2024)
*E. coli* ATCC 8739	[Tbp][Hex]	Stirring at room temperature	MIC: 5 mmol/L MBC: 5 mmol/L	(Panić et al. 2024)
*E. coli* ATCC 8739	[Bmim][Ben]	Anion exchange/Potentiometric titration	MIC: < 3.5 mmol/L MBC: < 3.5 mmol/L	(Cakó Bagány et al. 2024)
*E. coli* ATCC25922	CABILs: [Ch][Lys] [Ch][Arg] [Ch][His]	Heating and stirring	No inhibition zone	(Gao et al. 2024b)
*E. coli* CIP 54.8	TPA-P derivatives	Microwave-assisted	MIC: 16 mg/L	(Brunel et al. 2018)
*E. coli* CTX M2	[C_2_OHMIM][seco-Amx]	Stirring/ion-exchange	Broth micro dilution: MIC > 5 mmol/L	(Ferraz et al. 2020)
*E. coli* CTX M9	[C_2_OHMIM][seco-Amx]	Stirring/ion-exchange	Broth micro dilution: MIC > 5 mmol/L	(Ferraz et al. 2020)
*E. coli* DH5α	PBIC	Hydrothermal	MIC:32 µg/mL	(Zhou et al. 2024)
*E. coli* MTCC 40	C_10_mimCl	Synergistic interaction	MIC: 62500 µmol/L	(Siddiquee et al. 2021)
*F. nucleatum* ATCC25586	CAGE- IL	Hydrothermal	MIC:0.625 μg/mL MBC:2.5 μg/mL	(Yan et al. 2024)
*K. pneumoniae* ATCC 13883	Poly([C_12_VIm][Br])	Solvothermal	MIC:0.045 mg/mL	(Rackov et al. 2024)
*K. pneumoniae* ATCC 700603	[DC4][dicamba]_2_	Metathesis reaction	MIC: 1.442 mmol/L	(Wojcieszak et al. 2024)
*K. pneumoniae*	1,4phenylenebis(methanylylidene))bis(azanylydiene)bis(propane-3,1-diyl)bis(3- decyl-1H-imidazol-3-ium) bis bromide (3 g)	Solvothermal/Schiff base	MIC: 9.8 µg/mL	(ünver et al. 2024)
*K. pneumoniae* CIP 82.91	TPA-P derivatives	Microwave-assisted	MIC: 16 mg/L	(Brunel et al. 2018)
*K. pneumoniae* K6/ESBL ATCC 700603	Triphenyl(tetradecyl)phosphonium bromide	Quaternization	MIC: 8 µg/mL	(Cagide et al. 2024)
Multi drug resistant *A. baumannii*	PPh_3_C_12_–Br	Solvothermal	Inhibition zone 41.8 ± 0.9 mm MIC: 6.25 µmol/L	(Metelytsia et al. 2022)
*Porphyromonas gingivalis* ATCC33277	CAGE- IL	Hydrothermal	MIC:1.25 μg/mL MBC:2.5 μg/mL	(Yan et al. 2024)
*Porphyromonas gingivalis* W83	CAGE- IL	Hydrothermal	MIC:1.25 μg/mL MBC:2.5 μg/mL	(Yan et al. 2024)
*Prevotella intermedia* ATCC25611	CAGE- IL	Hydrothermal	MIC:1.25 μg/mL MBC:2.5 μg/mL	(Yan et al. 2024)
*P. mirabilis* ATCC 12453	[Tbp][Dodec]	Stirring at room temperature	MIC: 2.5 mmol/L MBC: 2.5 mmol/L	(Panić et al. 2024)
*P. mirabilis* ATCC 29906	Poly([C_12_VIm][Br])	Solvothermal	MIC:0.09 mg/mL	(Rackov et al. 2024)
*P. aeruginosa*	1,4phenylenebis(methanylylidene))bis(azanylydiene)bis(propane-3,1-diyl)bis(3- decyl-1H-imidazol-3-ium) bis bromide (3 g)	Solvothermal/Schiff base	MIC: 2.4 µg/mL	(ünver et al. 2024)
*P. aeruginosa* ATCC 27853	Triphenyl(tetradecyl)phosphonium bromide	Quaternization	MIC: 8 µg/mL	(Cagide et al. 2024)
*P. aeruginosa* ATCC 27853	[DC4][dicamba]_2_	Metathesis reaction	MIC: 0.715 mmol/L	(Wojcieszak et al. 2024)
*P. aeruginosa* ATCC 9027	[Bmim][Ben]	Anion exchange/Potentiometric titration	MIC: < 3.5 mmol/L MBC: < 3.5 mmol/L	(Cakó Bagány et al. 2024)
*P. fluorescens*	C14mimBF_4_	The alkylation of the MIM ring and metathesis reactions	MIC: 9.16 μg/mL MBC: 45.79 μg/mL	(Novello et al. 2024)
*P. fluorescens* ATCC 13525	Choline-based ILs	NR	NR	(Rita Pereira et al. 2022)
*P. putida* ATCC 49128	[DC4][dicamba]_2_	Metathesis reaction	MIC: 0.023 mmol/L	(Wojcieszak et al. 2024)
*P.. aeruginosa* ATCC 27853	PVA/CA/(C_16_MImCl) fibers	Solvothermal	Plate count: reduction in cell viability 69.42%	(P Libel et al. 2024)
*P.. aeruginosa* CIP 100,720	TPA-P derivatives	Microwave-assisted	MIC: 32 mg/L	(Brunel et al. 2018)
*Y. pseudotuberculosis*	1,4phenylenebis(methanylylidene))bis(azanylydiene)bis(propane-3,1-diyl)bis(3- decyl-1H-imidazol-3-ium) bis bromide (3 g)	Solvothermal/Schiff base	MIC: 2.4 µg/mL	(ünver et al. 2024)

*Fusobacterium nucleatum, F. nucleatum*; *NR*, not reported; *MIC*, minimum inhibitory concentration; *MBC*, minimum bactericidal concentration; *AWD*, agar well-diffusion; *[DC4][dicamba]*_*2*_, dicationic 3,6-dichloro-2-methoxy)benzoic acid; *TPA-P*, triphenylamine phosphonium; *[Tbp][Dodec]*, tetrabutylphosphonium dodecanoate; *CAGE*, choline and geranate; bromide, *[C*_*12*_*VIm][Br]*, 1-alkyl-3-vinylimidazolium bromide; *(P(VDF-TrFE)*, films of poly(vinylidene fluoride-trifluoroethylene); *[Emim][HSO*_*4*_*]*, 1-ethyl-3- methyl imidazolium hydrogen sulfate; *RTLMCs*, ruthenium-based low molecular weight chitosans; *C₁₄mimBF₄*, 1-tetradecyl-3-methylimidazolium tetrafluoroborate; *PBIC*, poly(butyl imidazole hexanoate); *PVA*, poly(vinyl alcohol); *CA*, citric acid; *CABILs*, choline-based amino acid ionic liquids; [Ch][Lys], choline lysinate; [Ch][Arg], choline argininate; [Ch][His], choline histidinate; *[C*_*2*_*OHMIM][seco-Amx]*, 1-(2-hydroxyethyl)−3-methylimidazolium seco-amoxicillinate; *seco*, indicates ring-opened (β-lactam–opened) amoxicillin anion; *C12MPBr*, 1-dodecyl-3-methylpyridinium bromide; *C*_*10*_*mimCl*, 1-decyl-3-methylimidazolium chloride; *C16MImCl*,1-hexadecyl-3-methylimidazolium chloride; *[Bmim][Ben]*, 1-butyl-3-methylimidazolium benzoate; *PPh*_*3*_*C*_*12*_*–Br*, dodecyl(triphenyl)phosphonium bromide; *[TTP][HFl]*, trihexyltetradecyl phosphonium coupled with monoprotonated fluorescein; *[Tbp][Hex]*, tetrabutylphosphonium-hexanoate; *C14mimBF*_*4*_, 1-tetradecyl-3-methylimidazolium tetrafluoroborate; *CFU*, colony forming unit

#### Antifungal activity of different ILs and IL-based materials

The therapy of fungal illnesses is becoming more difficult by the development of antifungal medication resistance, which has made many readily available drug classes useless. Increased efflux pump activity, cellular stress response pathway activation, and drug target alteration or overexpression are the main causes of fungal resistance. Among the most alarming resistant fungi are *Trichophyton indotineae*, *Aspergillus fumigatus* (*A. fumigatus*), and *Candida auris* (*C. auris*). Additionally, the absence of appropriate targets and the limited activity spectrum of conventional antifungal medications are drawbacks. New substitutes with potential antifungal activities have been created to overcome these restrictions, such as ILs and IL-based compounds (Bedair et al. 2025). In the study of ünver and his coworkers (ünver et al. 2024), bis-Schiff bases with imidazole (2) and bis-imidazolium liquids (3a-g) were produced. Their antimicrobial activity was evaluated toward a panel of different microorganisms, including four Gram-positive bacteria (*B. cereus*, *S. aureus*, *E. faecalis*, *and B. subtilis*), four Gram-negative bacteria (*Yersinia pseudotuberculosis* (*Y. pseudotuberculosis*), *P. aeruginosa*, *E. coli*, and *K. pneumoniae*), and two yeast-like fungi (*C. albicans* and *C. tropicalis*). The MICs were minimum in the case of 1,4 phenylenebis(methanylylidene))bis(azanylydiene)bis(propane-3,1-diyl)bis(3-decyl-1H-imidazol-3-ium) bis bromide (3 g) since it was 4.9 µg/mL for both spp. (ünver et al. 2024).

**Fig. 4:**
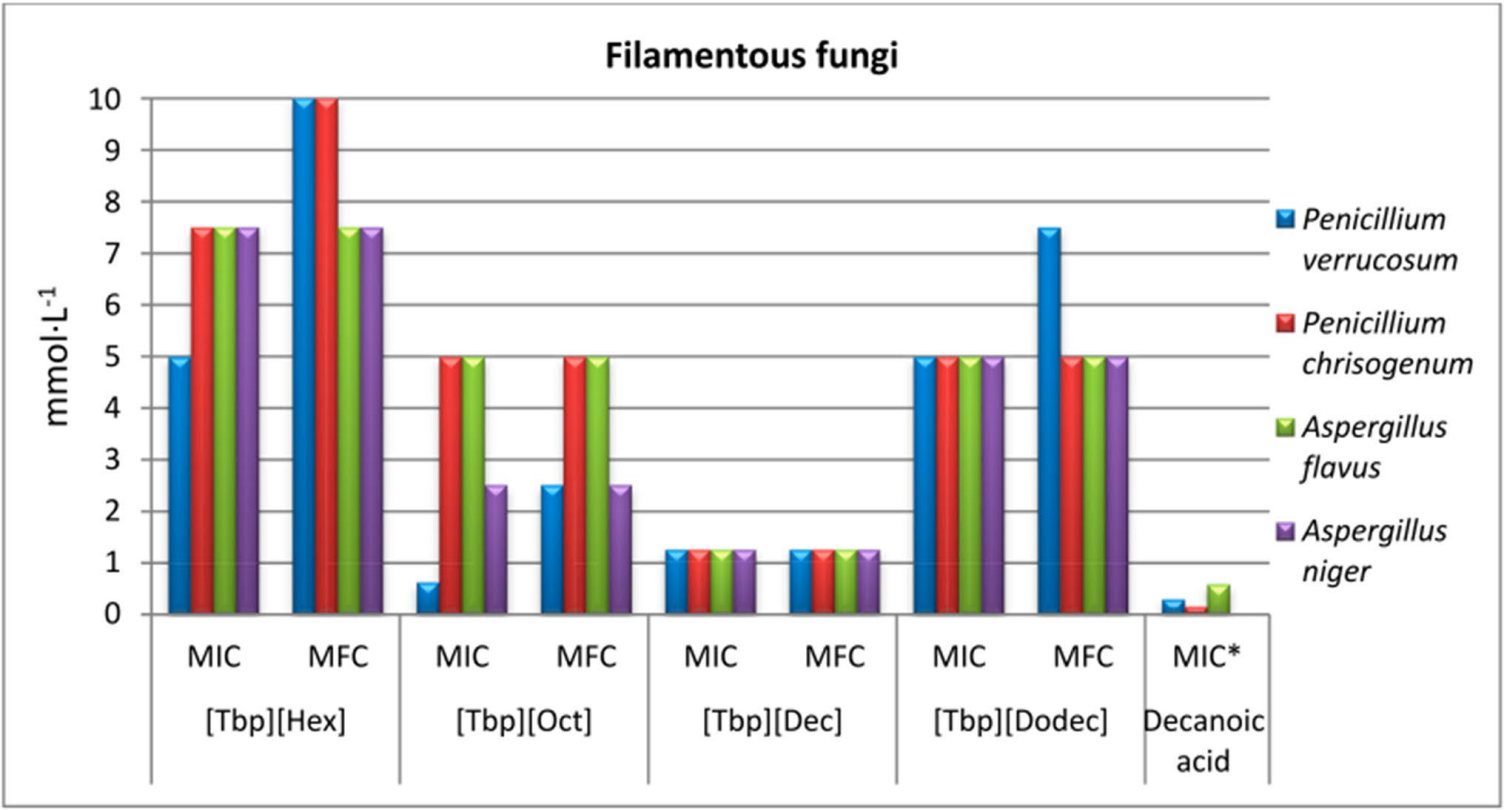
Antifungal effects of the four synthesized tetrabutylphosphonium ([Tbp]) ionic liquids incorporating hexanoate ([Hex]), octanoate ([Oct]), decanoate ([Dec]), and dodecanoate ([Dodec]) anions compared to decanoic acid as a comparative standard. The x-axis represents the evaluated ILs and the biological parameters used to assess their antifungal activity: minimum inhibitory concentration (MIC) and minimum fungicidal concentration (MFC); while the y-axis represents the corresponding concentration in mmol/L. With permission from Panić et al. (2024)

In another study of Cakó Bagány and his coworkers (Cakó Bagány et al. 2024), three ILs with 1-butyl-3-methylimidazolium cation and various carboxylate anions (phenylacetate, benzoate, and 4-methoxyphenylacetate) were produced and fully characterized. The antimicrobial activity of the developed ILs was evaluated against two Gram-negative bacteria (*E. coli* and *P. aeruginosa*) and three fungi (*Penicillium verrucosum (P. verrucosum*), *A. flavus*, and *A. parasiticus*) strains. The results showed that in comparison to the individual components, there was an improvement in the antimicrobial activity in the case of 1-butyl-3- methylimidazolium benzoate, which showed the highest lipophilicity and the lowest water solubility among the studied ILs, as shown in Fig. 4.

**Fig. 5:**
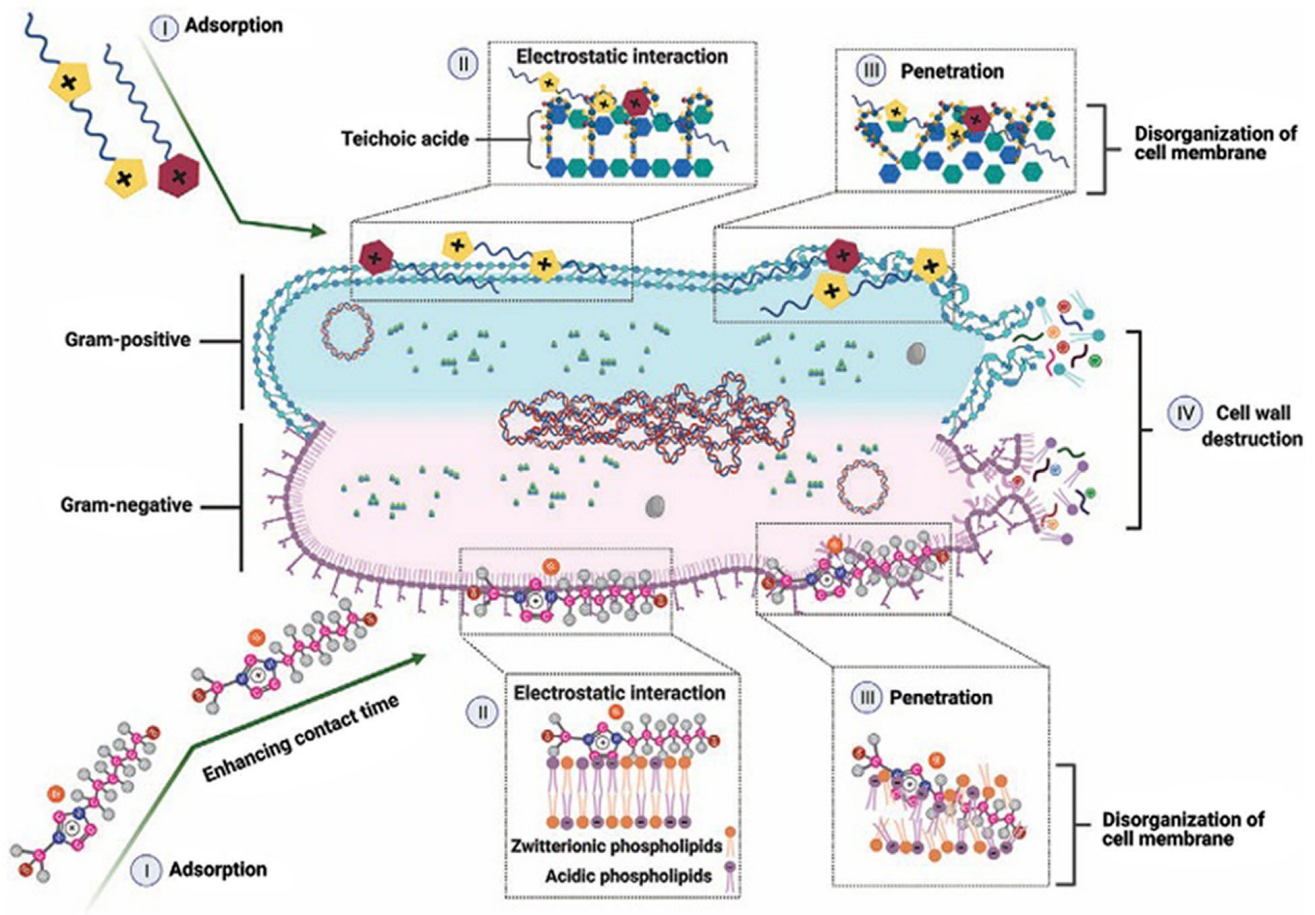
Mechanism of bacterial cell wall disruption by ILs in Gram-negative and Gram-positive bacteria through four sequential stages: (I) adsorption, the approach and accumulation of ILs at the bacterial cell wall/membrane; (II) electrostatic interactions between ILs and functional groups of the zwitterionic phospholipid bilayer; (III) penetration, insertion of ILs into the membrane causing structural disorganization; and (IV) cell wall destruction resulting in cell lysis. Reproduced with permission from the original source (Nikfarjam et al. 2021), under the Creative Commons Attribution (CC BY 4.0) license

Furthermore, in the study of Panić and his coworkers (Panić et al. 2024), tetrabutylphosphonium-hexanoate, -octanoate, -decanoate, and -dodecanoate were produced as potential antimicrobial ILs. Additionally, quaternary phosphonium-based ILs with fatty acid anions have shown great potential in this regard. The antimicrobial activity against various pathogens was determined, including three Gram-negative and Gram-positive bacteria, two yeasts, and four filamentous fungal strains. The results showed that among the most promising ILs towards the tested yeast, *C. guillermondii* and all tested filamentous fungi were tetrabutylphosphonium-decanoate (Fig. 5). The antifungal activities could be attributed to the presence of fatty acids and their derivatives against a variety of fungal spp. by introducing themselves into the lipid bilayers of fungal membranes, which compromised membrane integrity (Reddy and Nancharaiah 2020; Wang et al. 2020; Rackov et al. 2024; Wojcieszak et al. 2024). Table 3 shows more detailed information about the antifungal activities of various ILs and their derivatives.

**Table 3:** Antifungal activity of different ILs and ILs-based materials

**Tested microorganism**	**ILs/ILs-hybrid**	**Method of preparation**	**Antifungal assay**	**Reference**
A. *flavus* ATCC 9170	[C_12_VIm][Br]	Solvothermal	MIC:0.02 mg/mL	(Rackov et al. 2024)
*A. flavus* FCB0046	[Tbp][Dec]	Stirring at room temperature	MIC: 1.30 mmol/L MFC: 1.30 mmol/L	(Panić et al. 2024)
*A. flavus* FCB0046	[Bmim][Ben]	Anion exchange/Potentiometric titration	MIC:56.3 mmol/L MFC:56.3 mmol/L	(Cakó Bagány et al. 2024)
*A. niger* ATCC 16888	[C_12_VIm][Br]	Solvothermal	MIC:0.01 mg/mL	(Rackov et al. 2024)
*A. niger* FCB0046	[Tbp][Dec]	Stirring at room temperature	MIC: 1.30 mmol/L MFC: 1.30 mmol/L	(Panić et al. 2024)
*A. parasiticus* FCD0050	[Bmim][Ben]	Anion exchange/Potentiometric titration	MIC:56.3 mmol/L MFC:56.3 mmol/L	(Cakó Bagány et al. 2024)
*C. albicans*	1,4phenylenebis(methanylylidene))bis(azanylydiene)bis(propane-3,1-diyl)bis(3- decyl-1H-imidazol-3-ium) bis bromide (3 g)	Solvothermal/Schiff base	MIC: 4.9 µg/mL	(ünver et al. 2024)
*C. albicans* ATCC 10231	[Tbp][Dec]	Stirring at room temperature	MIC: 2.5 mmol/L MFC: 5 mmol/L	(Panić et al. 2024)
*C. albicans* ATCC 90028	Dodecyltriphenyl phosphonium bromide	Quaternization	MIC: less than/equal 0.25 µg/mL	(Cagide et al. 2024)
*C. albicans* ATCC 90028	[DC4][dicamba]_2_	Metathesis reaction	MIC:0.006 mmol/L	(Wojcieszak et al. 2024)
*C. albicans* ATCC10231	[C_16_MIM][Cl]	NR	MIC:4.68 µmol/L MFC:6.25 µmol/L	(Reddy and Nancharaiah 2020)
*C. albicans* clinical isolate i16	[C_16_MIM][Cl]	NR	MIC: 4.68 µmol/L MFC:6.25 µmol/L	(Reddy and Nancharaiah 2020)
*C. albicans* clinical isolate i21	[C_16_MIM][Cl]	NR	MIC: 9.38 µmol/L MFC:12.5 µmol/L	(Reddy and Nancharaiah 2020)
*C. albicans* MCCCF 98001	[VBIm][Br]	Solvothermal	Colony assay: no fungal growths with PMAV3	(Wang et al. 2020)
*C. guillermondii* JR-23	[Tbp][Dec]	Stirring at room temperature	MIC: 0.60 mmol/L MFC: 1.30 mmol/L	(Panić et al. 2024)
*C. tropicalis*	1,4phenylenebis(methanylylidene))bis(azanylydiene)bis(propane-3,1-diyl)bis(3- decyl-1H-imidazol-3-ium) bis bromide (3 g)	Solvothermal/Schiff base	MIC: 4.9 µg/mL	(ünver et al. 2024)
*M. mucedo* ATCC 20094	[C_12_VIm][Br]	Solvothermal	MIC:0.041 mg/mL	(Rackov et al. 2024)
*P. chrysogenum* FCB0035	[Tbp][Dec]	Stirring at room temperature	MIC: 1.30 mmol/L MFC: 1.30 mmol/L	(Panić et al. 2024)
*P. italicum* ATCC 10454	[C_12_VIm][Br]	Solvothermal	MIC:0.02 mg/mL	(Rackov et al. 2024)
*P. verrucosum* FCD0025	[Tbp][Oct]	Stirring at room temperature	MIC: 0.60 mmol/L MFC: 2.5 mmol/L	(Panić et al. 2024)
*P. verrucosum* FCD0025	[Bmim][Ben]	Anion exchange/Potentiometric titration	MIC:14.1 mmol/L MFC: 14.1 mmol/L	(Cakó Bagány et al. 2024)
*T. mentagrophytes* ATCC 9533	[C_12_VIm][Br]	Solvothermal	MIC:0.01 mg/mL	(Rackov et al. 2024)

*Mucor mucedo, M. mucedo*;* Trichophyton mentagrophytes*,* T. mentagrophytes*; *MIC*, minimum inhibitory concentration; *MFC*, minimum fungicidal concentration; *Fusobacterium nucleatum, F. nucleatum*; *NR*, not reported; *[DC4][dicamba]*_*2*_, dicationic 3,6-dichloro-2-methoxy)benzoic acid; *[C*_*12*_*VIm][Br]*, 1-alkyl-3-vinylimidazolium bromide; *[Tbp][Oct]*, tetrabutylphosphonium octanoate; *[Tbp][Dec]*, tetrabutylphosphonium decanoate; *C16MImCl*, 1-hexadecyl-3-methylimidazolium chloride; *[Bmim][Ben]*, 1-butyl-3- methylimidazolium benzoate; *ADD*, agar disk-diffusion

#### ILs as antibiofilm agents

**Fig. 6:**
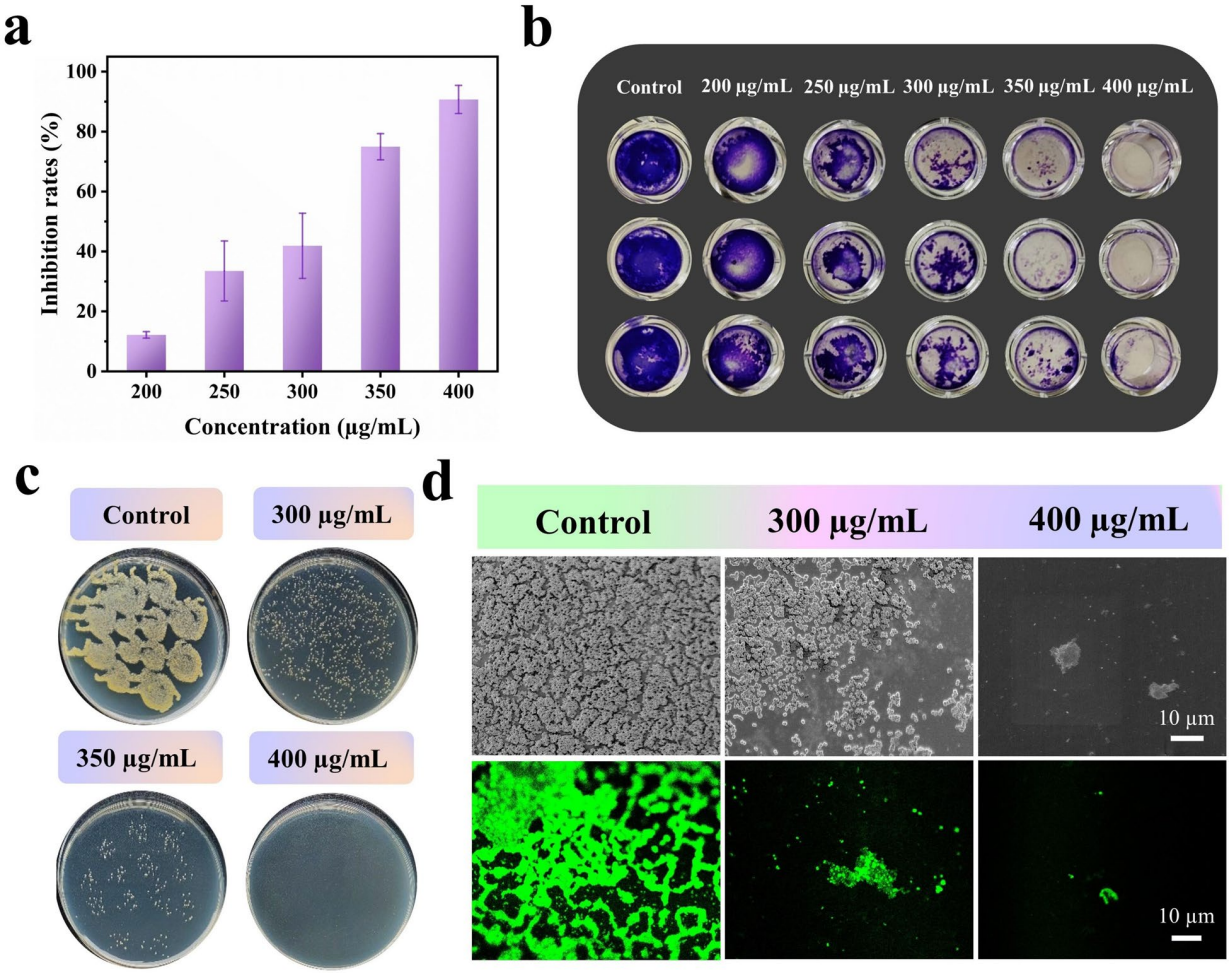
In vitro anti-biofilm activity of VOIMBr-CD. **a** Inhibition of *S. aureus* biofilms at varying VOIMBr-CD concentrations, measured by crystal violet assay. **b** Representative crystal violet-stained images and **c** corresponding plate images of *S. aureus* biofilms under different VOIMBr-CD concentrations. **d** SEM (top) and CLSM (bottom) pictures of *S. aureus* biofilms exposed to varying concentrations of VOIMBr-CD. Control refers to *S. aureus* biofilms that were not treated with VOIMBr-CD. Reproduced with permission from Gao et al. (2024a)

Bacterial biofilms are structured, three-dimensional microbial communities embedded within an extracellular polymeric substance (EPS) matrix composed mainly of polysaccharides, proteins, and extracellular deoxyribonucleic acid (DNA), which enables strong surface attachment and collective survival. Biofilm formation is clinically significant, as approximately 65–80% of bacterial infections are biofilm-associated and are often chronic and difficult to eradicate (Lebeaux et al. 2013; Bjarnsholt 2013). One of the principal challenges in treating biofilm-related infections is the markedly increased tolerance of biofilm-embedded bacteria to antimicrobial agents, with resistance levels reported to be up to 1000-fold higher than those of planktonic cells (Kalia et al. 2019). Biofilm formation and maintenance are regulated by complex signaling networks, including quorum sensing (QS) and the second messenger cyclic di-guanosine monophosphate, which collectively control adhesion, EPS production, and the transition from planktonic to sessile growth (Kalia et al. 2023). Among these pathways, QS systems are considered particularly attractive therapeutic targets, as their disruption can inhibit biofilm development and promote biofilm dispersal, thereby restoring bacterial susceptibility to antimicrobial agents (Kalia et al. 2019, 2023). In this context, ILs have emerged as promising antibiofilm agents. Several studies have demonstrated the ability of ILs to inhibit biofilm formation and disrupt mature biofilms formed by both Gram-positive and Gram-negative bacteria, including *B. cereus*, *S. aureus*, *P. fluorescens*, and *E. coli* (Rita Pereira et al. 2022; Zhou et al. 2024; Gao et al. 2024a). Mechanistically, ILs primarily exert antibiofilm activity through EPS disruption, increased membrane permeability, and damage to the bacterial cell wall, ultimately leading to biofilm destabilization and enhanced antimicrobial efficacy (Dupont and Suarez 2006). These findings demonstrate the growing potential of ILs as effective agents for combating biofilm-associated infections. Figure 6 shows the in vitro anti-biofilm activity of VOIMBr-CD using scanning electron microscopy (SEM) and confocal laser scanning microscopy (CLSM) against *S. aureus* at different concentrations of VOIMBr-CD (Gao et al. 2024a). It was found that the most frequent effects of ILs on biofilms and associated cells are biofilm disintegration (mostly EPS disruption and removal) coupled with peptidoglycan cell wall cleavage and changes in membrane permeabilization (Dupont and Suarez 2006).

### Antimicrobial in vivo activity of ILs

ILs demonstrate promising in vivo antimicrobial activity across animal models, functioning as effective antibacterial, antifungal, and antibiofilm agents (Pendleton and Gilmore 2015). Their mechanisms primarily involve disrupting microbial membranes, inducing oxidative stress, and enhancing penetration into complex structures. Studies in mice and rats show efficacy against pathogens such as *S. aureus*, *E. coli*, *P. aeruginosa*, and various fungal strains, often with low accompanying toxicity to host tissues. For instance, bis-phosphonium ILs like Di-Hex C10 have shown potent activity in mouse corneal wound models, clearing infections caused by MRSA and other ocular pathogens rapidly and with minimal cytotoxicity (O’Toole et al. 2012). The structural configuration of these compounds appears crucial for selectivity, as analogs with different designs proved toxic to host cells. Similarly, imidazole chloride ILs such as 1-dodecyl-3-methylimidazolium chloride demonstrated strong antibacterial effects in mouse skin abscess models by inducing internal oxidative stress and externally destroying the bacterial cell membrane (Hu et al. 2023). The length of the alkyl chain in these compounds correlates with their potency, with longer chains enhancing antimicrobial activity. Diketopyrrolopyrrole-based IL derivatives have shown significant therapeutic effects in rat wound models infected with *P. aeruginosa* (Zheng et al. 2020). Their efficacy depends on molecular size, where smaller derivatives penetrate the bacterial membrane to cause internal disruption, while larger ones intercalate into the lipid bilayer to destabilize it. This size-dependent activity directly influences healing outcomes. Another innovative approach involves chlorine ILs, which exhibit a dual-mode antibacterial action in mouse wound models (Wang et al. 2019). The cationic component binds to peptidoglycan, while the anion becomes activated in the acidic infection microenvironment, together achieving a substantial increase in potency compared to conventional treatments and promoting faster wound closure with good biocompatibility. Chiral imidazolium salts have shown selective activity against MRSA in toxicity screening models using *Galleria mellonella* (*G. mellonella*) larvae, a system that mimics mammalian responses (Coleman et al. 2012; Piatek et al. 2021). In a similar context, the in vivo experiments showed that VOIMBr-CD significantly relieved inflammation, facilitated collagen deposition and angiogenesis, which effectively accelerated wound healing in MRSA-infected mice models (Gao et al. 2024a). These findings emphasized their potential as therapeutic candidates for diseases like MRSA infection-like illnesses. The previous work presented an innovative approach by utilizing IL-based materials as emerging effective antibacterials, thereby raising awareness of the application prospects of IL-derived materials.

Antifungal activity is also evident in vivo, where ILs disrupt fungal membranes and inhibit ergosterol biosynthesis. In mouse models of maxillofacial defects, IL-loaded calcium phosphate bionanocomposites inhibited *Candida *spp. growth while also reducing inflammation and promoting tissue regeneration (Raucci et al. 2018). The length of the alkyl chains on the IL influenced the material’s properties and its antimicrobial effectiveness. Coumarin-pyrimidine IL derivatives have shown equipotent activity to standard antifungal drugs like miconazole in *G. mellonella* models infected with *A. fumigatus* and *C. albicans* (Tiwari et al. 2018). These compounds work by blocking ergosterol production and have demonstrated safety in both cellular and acute oral toxicity studies in mice. Related coumarin derivatives synthesized via IL catalysis have shown similar promise in mouse models against *C. albicans* with non-cytotoxic profiles (Tiwari et al. 2017).

The antibiofilm activity of ILs emerges from their ability to penetrate the extracellular matrix, disrupt QS, and destabilize the biofilm structure (Riduan and Zhang 2013; Liu et al. 2019). In rat wound models, certain IL derivatives dispersed *P. aeruginosa* biofilms effectively through membrane destabilization (Zheng et al. 2020). In mouse models, ILs integrated with nanotechnology, such as those using photodynamic activation, have shown synergistic effects in clearing stubborn biofilms formed by *E. coli* and *S. aureus*, thereby accelerating wound healing (Wang et al. 2019; Eleraky et al. 2020). Furthermore, biodegradable imidazolium salts have successfully inhibited biofilm formation by pathogens like *S. aureus* and *Listeria* *monocytogenes* (*L. monocytogenes*) in invertebrate infection models, highlighting their potential for selective disruption without environmental persistence (Coleman et al. 2012; Piatek et al. 2021).

### Clinical aspects, toxicity, and biodegradability of ILs

Despite some clear advantages in industrial applications and their frequent labeling as “green” owing to their non-volatility, some of the other properties of ILs are worth mentioning. Labeling this class of compounds either “green” or “toxic” represents a gross overgeneralization, which is neither warranted nor accurate (Petkovic et al. 2011). Numerous ILs exhibit wide-ranging toxicity and, in some cases, have been shown to be more toxic than the solvents for which they are potential replacements (Docherty and Kulpa 2005). ILs based on imidazole (most commonly the 1-alkyl-3-methylimidazolium chlorides ([C_*n*_mim]Cl) were among the first classes of IL to find applications on an industrial scale, most promisingly in cellulose processing (Fort et al. 2007). For this reason, a large proportion of studies conducted on the biological effects and potential environmental impact of ILs have focused on this group of compounds. [C_*n*_mim]Cls exhibit toxic effects against numerous biological test organisms and systems, both in vitro and in vivo, including enzymes, mammalian cells, luminescent marine bacteria, green algae, wheat, cress, duckweed, the soil invertebrate *Folsomia candida* (Matzke et al. 2007), the nematode *Caenorhabditis elegans* (Wu et al. 2013), earthworms (Luo et al. 2010), and zebrafish (Pretti et al. 2006), generally observable as a reduction in growth, viability, or reproduction, and have also been shown to cause malformations in mice as a result of prenatal exposure, suggesting a teratogenic effect (Bailey et al. 2008). As mentioned above, several animal and invertebrate models have been used to probe IL toxicity. Interestingly, in the study of Megaw and his coworkers, an alternative model in the form of the larvae of the Greater Wax Moth *G. mellonella* was used to study the toxicity of ILs (Megaw et al. 2015). The results showed that when *G. mellonella* larvae were exposed to a series of [C_*n*_mim]Cl, there was a clear increase in acute toxicity after a 24 h median lethal dose with increasing alkyl chain length and a characteristic “cut-off” effect, indicating strong structure–toxicity relationships in vivo (Megaw et al. 2015). Silkworm larvae similarly exhibited growth inhibition and mortality after exposure to imidazolium and pyridinium ILs, with median lethal concentration values depending on the types of anions and cations. It was found that oxidative-stress markers (superoxide dismutase, catalase, peroxidase, and malondialdehyde) were increased significantly to form an active protective mechanism for alleviating the toxic effects of ILs, as indicated by lipid peroxidation and cellular damage (Gao et al. 2021). Additionally, zebrafish exposed to task-specific imidazolium ILs showed acute toxicity after 96 h median lethal concentration in the 10^2^–10^3^ mg/L range and, at sub-lethal concentrations, elevated ROS and malondialdehyde, altered antioxidant enzymes (superoxide dismutase, catalase, glutathione S-transferase), and DNA damage in the liver, again linking IL exposure to oxidative stress and genotoxicity in vivo (Li et al. 2020). It was observed that functionalization (e.g., hydroxyethyl groups) can reduce toxicity compared with non-functionalized analogues (Li et al. 2020; Zhang et al. 2022). For rodents (mice, rats), it was revealed that mice receiving methylimidazolium ILs in drinking water for 18 weeks displayed only mild liver and kidney changes but marked alterations in gut microbiota composition, with increased *Lachnospiraceae*, *Clostridia*, and *Coriobacteriaceae* and shifts in xenobiotic/amino-acid metabolism pathways, suggesting microbiome disruption occurs before overt organ pathology (Young et al. 2020). Broader reviews summarize additional rodent studies, including intraperitoneal C8mim-based ILs causing kidney and liver pathology, with kidneys identified as a key target organ and a general trend of increasing toxicity with longer alkyl chains (Kumari et al. 2020; Cho et al. 2021; Gonçalves et al. 2021). Furthermore, a 2025 Nature Communications study shows in vivo that ILs with long cationic chains accumulate in mitochondria, induce mitophagy and apoptosis, and are tolerated 30–80-fold less than short-chain analogues across oral, intramuscular, and intravenous routes (Xing et al. 2025).

In the last years, ILs and antibiotic-based ILs are already proposed as tools to improve solubility, permeability, control of polymorphism, and bioavailability of classic antibiotics, and are being explored as topical agents, drug-delivery platforms, and antifouling/antibiofilm coatings (Ferraz et al. 2014; Miskiewicz et al. 2018; Prudêncio et al. 2020; Nikfarjam et al. 2021). Third-generation antibiotic-based ILs and OSILs based on β-lactams, fluoroquinolones, and streptomycin are specifically positioned as ways to recycle old antibiotics and target resistant pathogens (MRSA, multidrug-resistant Enterobacteriaceae, and uropathogenic *E. coli*) (Ferraz et al. 2014, 2020; Prudêncio et al. 2020; Costa et al. 2023). It was found that IL toxicity is highly structure-dependent since cation type and alkyl chain length markedly affect cytotoxicity, hemolysis, and biocompatibility (Ferraz et al. 2014; Miskiewicz et al. 2018; Nikfarjam et al. 2021; Hafeez et al. 2024; Liu et al. 2025). Additionally, long-chain cations often have strong antimicrobial but higher cytotoxic and hemolytic activity, with a “cut-off” effect at very long chains (Ferraz et al. 2014; Prudêncio et al. 2020; Nikfarjam et al. 2021). Some antibiotic-based OSILs show acceptable biocompatibility in vitro (fibroblasts, keratinocytes, red blood cells) at concentrations close to their MICs, but toxicity increases at higher doses (Ferraz et al. 2014; Miskiewicz et al. 2018; Nikfarjam et al. 2021; Hafeez et al. 2024; Faísca et al. 2025; Liu et al. 2025). Biodegradability and aquatic toxicity are also strongly influenced by the cation and chain length since ILs with short alkyl chains (C1–C5) are generally more biodegradable and less toxic to aquatic organisms than those with long chains (≥ C7) (Ferraz et al. 2014; Prudêncio et al. 2020; Nikfarjam et al. 2021). Fluoroquinolone- and β-lactam-based OSILs can be tuned to retain or even increase activity against pathogens while reducing activity against some commensal strains, suggesting potential for narrower-spectrum or microbiota-sparing profiles (Costa et al. 2023). Conversely, broad-spectrum, highly potent ILs may also disrupt beneficial microbiota. Unfortunately, current data are almost entirely from pathogenic test strains, not full microbiome models.

## Conclusion

New materials with strong antibacterial and antifungal abilities must be discovered and developed in order to address the growing issue of antimicrobial resistance. This review has brought attention to the important potential of various ILs as antimicrobial and antibiofilm agents. Infections caused by bacteria and fungi pose a serious public health threat, especially for those with weakened immune systems. The therapy of these diseases has become even more challenging due to the rising incidence of antimicrobial medication resistance, highlighting the urgent need for innovative therapeutic approaches. This review discusses the potential of ILs as effective antimicrobial agents. Synthesized through diverse approaches, ILs have exhibited significant antifungal and antibacterial activities against a wide spectrum of microbial spp. Their performance, evaluated through parameters such as MIC, minimum bactericidal concentration, minimum fungicidal concentration, growth inhibition rate, and inhibition zone measurements, highlights their strong antimicrobial efficacy. In particular, ILs present distinct advantages over conventional antimicrobial agents, including customizable physicochemical properties, lower toxicity, and a reduced likelihood of resistance development. However, more extensive in vitro and in vivo studies are necessary to fully elucidate their mechanisms of action and assess their efficacy and safety profiles before clinical applications. With continued research, these substances could potentially serve as supplements or substitutes for traditional antimicrobial therapies in the future. The review’s conclusions highlight the value of ILs in enhancing public health outcomes and combating the worldwide conventional antimicrobial resistance challenge.

## Supplementary Information

Below is the link to the electronic supplementary material.

ESM1 (DOCX 24.5 KB)

## Data Availability

No datasets were generated or analyzed during the current study.

## Abbreviations

ADD: Agar disk-diffusion
AWD: Agar well-diffusion
CA: Citric acid
CABILs: Choline-based amino acid ionic liquids
CAGE: Choline and geranate
[C_12_VIm][Br]: 1-Alkyl-3-vinylimidazolium bromide
(C_14_mimBF_4_): 1-Tetradecyl-3-methylimidazolium tetrafluoroborate
[Ch][Arg]: Choline argininate
[Ch][His]: Choline histidinate
[Ch][Lys]: Choline lysinate
[Cho][Ala]: Cholinium alaninate
CFU: Colony-forming unit
CLSM: Confocal laser scanning microscope
Cu@PIL1.2-h: Heated copper-based nanoparticles within the polymeric ionic liquids
[C_2_OHMIM][seco-Amx]: 1-(2-Hydroxyethyl)-3-methylimidazolium seco-amoxicillinate
[DC_4_][dicamba]_2_: Dicationic 3,6-dichloro-2-methoxy)benzoic acid
[Dec]: Decanoate
[Dodec]: Dodecanoate
DNA: Deoxyribonucleic acid
[Emim][HSO4]: 1-Ethyl-3-methylimidazolium hydrogen sulfate
EPS: Extracellular polymeric substance
ILs: Ionic liquids
MBC: Minimum bactericidal concentration
MFC: Minimum fungicidal concentration
MIC: Minimum inhibitory concentration
MIM: Methyl imidazolium
MV: Microwave
OSILs: Organic salts ILs
[Oct]: Octanoate
(P(VDF-TrFE): Films of poly(vinylidene fluoride-trifluoroethylene)
PBIC: Poly(butyl imidazole hexanoate)
PPh3C12–Br: Dodecyl(triphenyl)phosphonium bromide
PMAV_3_: Poly(MMA-co-AM-co-[VBIm]Br)
MMA: Methyl methacrylate
AM: Acrylamide
[VBIm]Br: 1-Vinyl-3-butylimidazolium bromide
PVA: Poly(vinyl alcohol)
QS: Quorum sensing
ROS: Reactive oxygen species
RTLMCs: Ruthenium-based low molecular weight chitosans
SAILs: Surface-active ILs
SEM: Scanning electron microscope
Spp: Species
[Tbp][Hex]: Tetrabutylphosphonium-hexanoate
[TTP][HFl]: Trihexyltetradecyl phosphonium coupled with monoprotonated fluorescein
[TTP]2[Fl]: Trihexyltetradecyl phosphonium coupled with dianionic fluorescein
TPA-P: Triphenylamine phosphonium
US: Ultrasonic
VAN: Vancomycin
VOIMBr-CD: 1-Vinylimidazole, bromooctane-carbon dot
[CnMIM] + [X]-: 1-Alkyl-3-methyl imidazolium ionic liquids

## References

[CR1] Abdallah IA, Hammad SF, Bedair A, Abdelaziz MA, Danielson ND, Elshafeey AH, Mansour FR (2022) A gadolinium-based magnetic ionic liquid for supramolecular dispersive liquid–liquid microextraction followed by HPLC/UV for the determination of favipiravir in human plasma. Biomed Chromatogr 36:1–10. 10.1002/bmc.5365 10.1002/bmc.5365 35274347

[CR2] Abdelaziz MA, Saleh AM, Mansour FR, Danielson ND (2023) A gadolinium-based magnetic ionic liquid for dispersive liquid-liquid microextraction of ivermectin from environmental water. J Chromatogr Sci 31:988–994. 10.1093/chromsci/bmac101 10.1093/chromsci/bmac101 36533420

[CR3] Ahmed SK, Hussein S, Qurbani K, Ibrahim RH, Fareeq A, Mahmood KA, Mohamed MG (2024) Antimicrobial resistance: impacts, challenges, and future prospects. J Med Surg Public Health 2:100081. 10.1016/j.glmedi.2024.100081

[CR4] Bailey MM, Townsend MB, Jernigan PL, Sturdivant J, Hough-Troutman WL, Rasco JF, Swatloski RP, Rogers RD, Hood RD (2008) Developmental toxicity assessment of the ionic liquid 1-butyl-3-methylimidazolium chloride in CD-1 mice. Green Chem 10:1213–1217. 10.1039/B807019A

[CR5] Bedair HM, Hamed M, Mansour FR (2024) New emerging materials with potential antibacterial activities. Appl Microbiol Biotechnol 108:515. 10.1007/s00253-024-13337-6 39540988 10.1007/s00253-024-13337-6 PMC11564324

[CR6] Bedair HM, Samir TM, Mansour FR (2024) Antibacterial and antifungal activities of natural deep eutectic solvents. Appl Microbiol Biotechnol 108:198. 10.1007/s00253-024-13044-2 38324052 10.1007/s00253-024-13044-2 PMC10850035

[CR7] Bedair HM, Hamed M, Mansour FR (2025) Insight into new emerging materials as antifungal agents and delivery systems : a scoping review. J Drug Deliv Sci Technol 104:106499. 10.1016/j.jddst.2024.106499

[CR8] Binjawhar DN, Alfaifi MY, El MA, Shati AA, Eldin S, Elbehairi I, Fayad E, Abdellatif M, Elshaarawy RFM, Hassan A (2024) Upgrading the antibacterial and antibiofilm potential of nanoruthenium via encapsulation by thiazolium ionic liquids-functionalized chitosan film. Eur Polym J 207:112822. 10.1016/j.eurpolymj.2024.112822

[CR9] Bjarnsholt T (2013) The role of bacterial biofilms in chronic infections. APMIS 121:1–58. 10.1111/apm.12099 23635385 10.1111/apm.12099

[CR10] Brunel F, Lautard C, di Giorgio C, Garzino F, Raimundo JM, Bolla JM, Camplo M (2018) Antibacterial activities of mono-, di- and tri-substituted triphenylamine-based phosphonium ionic liquids. Bioorg Med Chem Lett 28:926–929. 10.1016/j.bmcl.2018.01.057 29439903 10.1016/j.bmcl.2018.01.057

[CR11] Busetti A, Crawford DE, Earle MJ, Gilea MA, Gilmore BF, Gorman SP, Laverty G, Lowry AF, McLaughlin M, Seddon KR (2010) Antimicrobial and antibiofilm activities of 1-alkylquinolinium bromide ionic liquids. Green Chem 12:420–425. 10.1039/B919872E

[CR12] Cagide F, Borges F, Sim M (2024) Antimicrobial activity and cytotoxicity of novel quaternary ammonium and phosphonium salts. 401. 10.1016/j.molliq.2024.124616

[CR13] Cakó Bagány N, Čapelja E, Kovačević S, Karaman M, Podunavac-Kuzmanović S, Gadžurić S, Belić S (2024) Experimental and in silico comparative study of physicochemical properties and antimicrobial activity of carboxylate ionic liquids. Molecules. 10.3390/molecules29153668 39125070 10.3390/molecules29153668 PMC11314197

[CR14] Campos PA De, Royer S, Brito CS De, Arau BF, Rosineide PPG, Ribas M (2016) Multidrug resistance related to biofilm formation in Acinetobacter baumannii and Klebsiella pneumoniae clinical strains from different pulsotypes. Curr Microbiol 617–627. 10.1007/s00284-016-0996-x 10.1007/s00284-016-0996-x 26846651

[CR15] Carvalho EO, Marques-Almeida T, Cruz BDD, Correia DM, Esperança JMSS, Irastorza I, Silvan U, Fernandes MM, Lanceros-Mendez S, Ribeiro C (2024) Piezoelectric biomaterials with embedded ionic liquids for improved orthopedic interfaces through osseointegration and antibacterial dual characteristics. Biomater Adv 164. 10.1016/j.bioadv.2024.213970 10.1016/j.bioadv.2024.213970 39106539

[CR16] Cho C-W, Pham TPT, Zhao Y, Stolte S, Yun Y-S (2021) Review of the toxic effects of ionic liquids. Sci Total Environ 786:147309. 10.1016/j.scitotenv.2021.147309 33975102 10.1016/j.scitotenv.2021.147309

[CR17] Coleman D, Špulák M, Garcia MT, Gathergood N (2012) Antimicrobial toxicity studies of ionic liquids leading to a ‘hit’ MRSA selective antibacterial imidazolium salt. Green Chem 14:1350. 10.1039/c2gc16090k

[CR18] Cornellas A, Perez L, Comelles F, Ribosa I, Manresa A, Garcia MT (2011) Self-aggregation and antimicrobial activity of imidazolium and pyridinium based ionic liquids in aqueous solution. J Colloid Interface Sci 355:164–171. 10.1016/j.jcis.2010.11.063 21186035 10.1016/j.jcis.2010.11.063

[CR19] Costa FMS, Granja A, Pérez RL, Warner IM, Reis S, Passos MLC, Saraiva MLMFS (2023) Fluoroquinolone-based organic salts (GUMBOS) with antibacterial potential. Int J Mol Sci 24. 10.3390/ijms242115714 10.3390/ijms242115714 PMC10650486 37958698

[CR20] Das S, Paul A, Bera D, Dey A, Roy A, Dutta A, Ganguly D (2021) Design, development and mechanistic insights into the enhanced antibacterial activity of mono and bis-phosphonium fluoresceinate ionic liquids. Mater Today Commun 28:102672. 10.1016/j.mtcomm.2021.102672

[CR21] Di Martino P (2022) Antimicrobial agents and microbial ecology. AIMS Microbiol 8:1–4. 10.3934/microbiol.2022001 35496989 10.3934/microbiol.2022001 PMC8995183

[CR22] Docherty KM, Kulpa JCF (2005) Toxicity and antimicrobial activity of imidazolium and pyridinium ionic liquids. Green Chem 7:185–189. 10.1039/B419172B

[CR23] Dupont J, Suarez PAZ (2006) Physico-chemical processes in imidazolium ionic liquids. Phys Chem Chem Phys 8:2441–2452. 10.1039/B602046A 16721427 10.1039/b602046a

[CR24] Eleraky NE, Allam A, Hassan SB, Omar MM (2020) Nanomedicine fight against antibacterial resistance: an overview of the recent pharmaceutical innovations. Pharmaceutics 12:142. 10.3390/pharmaceutics12020142 32046289 10.3390/pharmaceutics12020142 PMC7076477

[CR25] Faísca F, Santos AFM, Ferreira M, Gameiro P, Lima SAC, Branco LC (2025) Organic salts based on streptomycin: a new approach for an old drug. Mol Pharm 22:5361–5372. 10.1021/acs.molpharmaceut.5c00310 40812790 10.1021/acs.molpharmaceut.5c00310

[CR26] Ferraz R, Teixeira V, Rodrigues D, Fernandes R, Prudêncio C, Noronha JP, Petrovski Ž, Branco LC (2014) Antibacterial activity of ionic liquids based on ampicillin against resistant bacteria. RSC Adv 4:4301–4307. 10.1039/C3RA44286A

[CR27] Ferraz R, Silva D, Dias AR, Dias V, Santos MM, Pinheiro L, Prudêncio C, Noronha JP, Petrovski Ž, Branco LC (2020) Synthesis and antibacterial activity of ionic liquids and organic salts based on penicillin g and amoxicillin hydrolysate derivatives against resistant bacteria. Pharmaceutics 12. 10.3390/pharmaceutics12030221 10.3390/pharmaceutics12030221 PMC7150922 32131540

[CR28] Flieger J, Feder-Kubis J, Tatarczak-Michalewska M (2020) Chiral ionic liquids: structural diversity, properties and applications in selected separation techniques. Int J Mol Sci 21. 10.3390/ijms21124253 10.3390/ijms21124253 PMC7352568 32549300

[CR29] Florio W, Becherini S, D’Andrea F, Lupetti A, Chiappe C, Guazzelli L (2019) Comparative evaluation of antimicrobial activity of different types of ionic liquids. Mater Sci Eng C Mater Biol 104:109907. 10.1016/j.msec.2019.109907 10.1016/j.msec.2019.109907 31499958

[CR30] Fort DA, Remsing RC, Swatloski RP, Moyna P, Moyna G, Rogers RD (2007) Can ionic liquids dissolve wood? Processing and analysis of lignocellulosic materials with 1-n-butyl-3-methylimidazolium chloride. Green Chem 9:63–69. 10.1039/b607614a

[CR31] Gao K, Li B, Chen R, Qian P, Dong J, Xue C, Guo X (2021) Ecotoxicology and environmental safety a feasibility study of using silkworm larvae as a novel in vivo model to evaluate the biotoxicity of ionic liquids. Ecotoxicol Environ Saf 209:111759. 10.1016/j.ecoenv.2020.111759 33341695 10.1016/j.ecoenv.2020.111759

[CR32] Gao C, Qu X, Fu L, Chen J, Chu Y, Qiu H (2024a) Ionic liquid-derived carbon dots with enhanced antibacterial activity and antibiofilm capability for treatment of MRSA-infected wounds. Chem Eng J 497:154717. 10.1016/j.cej.2024.154717

[CR33] Gao X, He Q, Zhou G, Ali A, Yu C, Yao S (2024b) Choline and amino acids-based ionic liquids (CABILs) for the preparation of new antibacterial coating with loofah and epoxy resin. Ind Crops Prod 210:118093. 10.1016/j.indcrop.2024.118093

[CR34] Garcia MT, Ribosa I, González JJ, Comelles F (2020) Surface activity, self-aggregation and antimicrobial activity of catanionic mixtures of surface active imidazolium- or pyridinium-based ionic liquids and sodium bis(2-ethylhexyl) sulfosuccionate. J Mol Liq 303:112637. 10.1016/j.molliq.2020.112637

[CR35] García MT, Bautista E, de la Fuente A, Pérez L (2023) Cholinium-based ionic liquids as promising antimicrobial agents in pharmaceutical applications: surface activity, antibacterial activity and ecotoxicological profile. Pharmaceutics. 10.3390/pharmaceutics15071806 37513993 10.3390/pharmaceutics15071806 PMC10385515

[CR36] Gonçalves ARP, Paredes X, Cristino AF, Santos FJ V, Queirós CSGP (2021) Ionic liquids—a review of their toxicity to living organisms. Int J Mol Sci 22. 10.3390/ijms22115612 10.3390/ijms22115612 PMC8198260 34070636

[CR37] Hafeez S, Rasool Z, Hafeez S, Zafar R (2024) Imidazolium , pyridinium and pyrazinium based ionic liquids with octyl side chains as potential antibacterial agents against multidrug resistant uropathogenic E . coli. Heliyon 10:e39829. 10.1016/j.heliyon.2024.e39829 39634437 10.1016/j.heliyon.2024.e39829 PMC11616562

[CR38] Hu Y, Xing Y, Ye P, Yu H, Meng X, Song Y, Wang G, Diao Y (2023) The antibacterial activity and mechanism of imidazole chloride ionic liquids on *Staphylococcus aureus*. Front Microbiol 14:1109972. 10.3389/fmicb.2023.1109972 36814568 10.3389/fmicb.2023.1109972 PMC9939751

[CR39] Kalia VC, Patel SKS, Kang YC, Lee J-K (2019) Quorum sensing inhibitors as antipathogens: biotechnological applications. Biotechnol Adv 37:68–90. 10.1016/j.biotechadv.2018.11.006 30471318 10.1016/j.biotechadv.2018.11.006

[CR40] Kalia VC, Patel SKS, Lee J (2023) Ecotoxicology and environmental safety bacterial biofilm inhibitors : an overview. Ecotoxicol Environ Saf 264:115389. 10.1016/j.ecoenv.2023.115389 37634478 10.1016/j.ecoenv.2023.115389

[CR41] Kumari P, Pillai VVS, Benedetto A (2020) Mechanisms of action of ionic liquids on living cells: the state of the art. Biophys Rev 12(5):1187–1215. 10.1007/s12551-020-00754-w 10.1007/s12551-020-00754-w PMC7575683 32936423

[CR42] Lebeaux D, Chauhan A, Rendueles O, Beloin C (2013) From in vitro to in vivo models of bacterial biofilm-related infections. 288–356. 10.3390/pathogens2020288 10.3390/pathogens2020288 PMC4235718 25437038

[CR43] Li W, Zhu L, Du Z, Li B, Wang J, Wang J, Zhang C, Zhu L (2020) Acute toxicity, oxidative stress and DNA damage of three task-specific ionic liquids ([C2NH2MIm]BF4, [MOEMIm]BF4, and [HOEMIm]BF4) to zebrafish (*Danio rerio*). Chemosphere 249:126119. 10.1016/j.chemosphere.2020.126119 32044610 10.1016/j.chemosphere.2020.126119

[CR44] P Libel G, Facchi SP, de Almeida DA, Madruga LC, Kipper MJ, Schrekker HS, Martins AF, Radovanovic E (2024) Cross-linked poly(vinyl alcohol)/citric acid electrospun fibers containing imidazolium ionic liquid with enhanced antiadhesive and antimicrobial properties. Mater Chem Phys 316. 10.1016/j.matchemphys.2024.129087

[CR45] Liu Y, Shi L, Su L, van der Mei HC, Jutte PC, Ren Y, Busscher HJ (2019) Nanotechnology-based antimicrobials and delivery systems for biofilm-infection control. Chem Soc Rev 48:428–446. 10.1039/c7cs00807d 30601473 10.1039/c7cs00807d

[CR46] Liu X, Zhao X, Qiu H, Liang W, Liu L, Sun Y, Zhao L, Wang X, Liang H (2025) Antibacterial activity of GO-based composites enhanced by phosphonate-functionalized ionic liquids and silver. Materials. 10.3390/ma18081889 40333535 10.3390/ma18081889 PMC12028358

[CR47] Luo Y-R, San-Hu W, Li X-Y, Yun M-X, Wang J-J, Sun Z-J (2010) Toxicity of ionic liquids on the growth, reproductive ability, and ATPase activity of earthworm. Ecotoxicol Environ Saf 73:1046–1050. 10.1016/j.ecoenv.2010.01.017 20149456 10.1016/j.ecoenv.2010.01.017

[CR48] Martins-Santana L, Rezende CP, Rossi A, Martinez-Rossi NM, Almeida F (2023) Addressing microbial resistance worldwide: challenges over controlling life-threatening fungal infections. Pathogens 12:1–15. 10.3390/pathogens12020293 10.3390/pathogens12020293 PMC9961291 36839565

[CR49] Matzke M, Stolte S, Thiele K, Juffernholz T, Arning J, Ranke J, Welz-Biermann U, Jastorff B (2007) The influence of anion species on the toxicity of 1-alkyl-3-methylimidazolium ionic liquids observed in an (eco)toxicological test battery. Green Chem 9:1198–1207. 10.1039/B705795D

[CR50] Megaw J, Thompson TP, Lafferty RA, Gilmore BF (2015) *Galleria mellonella* as a novel in vivo model for assessment of the toxicity of 1-alkyl-3-methylimidazolium chloride ionic liquids. Chemosphere 139:197–201. 10.1016/j.chemosphere.2015.06.026 26121605 10.1016/j.chemosphere.2015.06.026

[CR51] Metelytsia LO, Hodyna DM, Semenyuta IV, Kovalishyn VV, Rogalsky SP, Derevianko YK, Brovarets VS, Tetko IV (2022) Theoretical and experimental studies of phosphonium ionic liquids as potential antibacterials of MDR Acinetobacter baumannii. Antibiotics 11. 10.3390/antibiotics11040491 10.3390/antibiotics11040491 PMC9025513 35453241

[CR52] Miralles-Comins S, Zanatta M, Embid SG, Alleva M, Chiappone A, Roppolo I, Mitchell SG, Sans V (2024) Development of high-resolution 3D printable polymerizable ionic liquids for antimicrobial applications. Device 2. 10.1016/j.device.2023.100224

[CR53] Miskiewicz A, Ceranowicz P, Szymczak M, Bartuś K, Kowalczyk P (2018) The use of liquids ionic fluids as pharmaceutically active substances helpful in combating nosocomial infections induced by Klebsiella Pneumoniae New Delhi strain, Acinetobacter Baumannii and Enterococcus species. Int J Mol Sci 19. 10.3390/ijms19092779 10.3390/ijms19092779 PMC6163946 30223584

[CR54] Nikfarjam N, Ghomi M, Agarwal T, Hassanpour M, Sharifi E, Khorsandi D, Ali Khan M, Rossi F, Rossetti A, Nazarzadeh Zare E, Rabiee N, Afshar D, Vosough M, Kumar Maiti T, Mattoli V, Lichtfouse E, Tay FR, Makvandi P (2021) Antimicrobial ionic liquid-based materials for biomedical applications. Adv Funct Mater 31. 10.1002/adfm.202104148

[CR55] Novello E, Scalzo G, D’Agata G, Raucci MG, Ambrosio L, Soriente A, Tomasello B, Restuccia C, Parafati L, Consoli GML, Ferreri L, Rescifina A, Zagni C, Zampino DC (2024) Synthesis, characterisation, and in vitro evaluation of biocompatibility, antibacterial and antitumor activity of imidazolium ionic liquids. Pharmaceutics. 10.3390/pharmaceutics16050642 38794304 10.3390/pharmaceutics16050642 PMC11125126

[CR56] O’Toole GA, Wathier M, Zegans ME, Shanks RMQ, Kowalski R, Grinstaff MW (2012) Diphosphonium ionic liquids as broad-spectrum antimicrobial agents. Cornea 31:810–816. 10.1097/ICO.0b013e31823f0a86 22236790 10.1097/ICO.0b013e31823f0a86 PMC3336019

[CR57] Panić JJ, Saletović M, Rakić M, Čapelja E, Janković N, Papović SM, Vraneš MB (2024) Biocompatible tetrabutylphosphonium-based ionic liquids with medium-chain fatty acids as anions: thermo-physical and antimicrobial profile. J Mol Liq 399. 10.1016/j.molliq.2024.124420

[CR58] Pendleton JN, Gilmore BF (2015) The antimicrobial potential of ionic liquids: a source of chemical diversity for infection and biofilm control. Int J Antimicrob Agents 46:131–139. 10.1016/j.ijantimicag.2015.02.016 25907139 10.1016/j.ijantimicag.2015.02.016

[CR59] Petkovic M, Seddon KR, Rebelo LPN, Pereira CS (2011) Ionic liquids: a pathway to environmental acceptability. Chem Soc Rev 40:1383–1403. 10.1039/c004968a 21116514 10.1039/c004968a

[CR60] Peyclit L, Yousfi H, Rolain J-M, Bittar F (2021) Drug repurposing in medical mycology: identification of compounds as potential antifungals to overcome the emergence of multidrug-resistant fungi. Pharmaceuticals 14:488. 10.3390/ph14050488 34065420 10.3390/ph14050488 PMC8161392

[CR61] Piatek M, Sheehan G, Kavanagh K (2021) *Galleria mellonella*: the versatile host for drug discovery, in vivo toxicity testing and characterising host-pathogen interactions. Antibiot 10:1545. 10.3390/antibiotics10121545 10.3390/antibiotics10121545 PMC8698334 34943757

[CR62] Pretti C, Chiappe C, Pieraccini D, Gregori M, Abramo F, Monni G, Intorre L (2006) Acute toxicity of ionic liquids to the zebrafish (Danio rerio). In: Green Chemistry. pp 238–240

[CR63] Prudêncio C, Vieira M, der Auweraer S, Ferraz R (2020) Recycling old antibiotics with ionic liquids. Antibiotics 9. 10.3390/antibiotics9090578 10.3390/antibiotics9090578 PMC7558273 32899785

[CR64] Rackov S, Pilić B, Janković N, Kosanić M, Petković M, Vraneš M (2024) From synthesis to functionality: tailored ionic liquid-based electrospun fibers with superior antimicrobial properties. Polymers (Basel) 16. 10.3390/polym16152094 10.3390/polym16152094 PMC11314316 39125121

[CR65] Rangarajan R, Venkataraman R (2020) Antibiotics targeting Gram-negative bacteria. Elsevier Inc

[CR66] Ratti R (2014) Ionic liquids: synthesis and applications in catalysis. Adv Chem 2014:1–16. 10.1155/2014/729842

[CR67] Raucci MG, Fasolino I, Pastore SG, Soriente A, Capeletti LB, Dessuy MB, Giannini C, Schrekker HS, Ambrosio L (2018) Antimicrobial imidazolium ionic liquids for the development of minimal invasive calcium phosphate-based bionanocomposites. ACS Appl Mater Interfaces 10:42766–42776. 10.1021/acsami.8b12696 30456941 10.1021/acsami.8b12696

[CR68] Reddy GKK, Nancharaiah YV (2020) Alkylimidazolium ionic liquids as antifungal alternatives: antibiofilm activity against *Candida albicans* and underlying mechanism of action. Front Microbiol 11:1–15. 10.3389/fmicb.2020.00730 32373105 10.3389/fmicb.2020.00730 PMC7186398

[CR69] Report S (2014) Antimicrobial resistance in the EU in 2012. Vet Rec 174:341 24700003 10.1136/vr.g2500

[CR70] Riduan SN, Zhang Y (2013) Imidazolium salts and their polymeric materials for biological applications. Chem Soc Rev 42:9055–9070. 10.1039/c3cs60169b 23979404 10.1039/c3cs60169b

[CR71] Rita Pereira A, Gomes IB, Simões M (2022) Choline-based ionic liquids for planktonic and biofilm growth control of *Bacillus cereus* and *Pseudomonas fluorescens*. J Mol Liq 346:117077. 10.1016/j.molliq.2021.117077

[CR72] Siddiquee MA, Saraswat J, Imtiyaz K, Bhat AR, Wani FA, Alanazi AM, Khan AA, Rizvi MMA, Patel R (2021) In-vitro cytotoxicity, synergistic antibacterial activity and interaction studies of imidazolium-based ionic liquids with levofloxacin. J Mol Liq 325:115125. 10.1016/j.molliq.2020.115125

[CR73] Singh SK, Savoy AW (2020) Ionic liquids synthesis and applications: an overview. J Mol Liq 297:112038. 10.1016/j.molliq.2019.112038

[CR74] Takahashi C, Hattori Y, Yagi S, Murai T, Tanemura M, Kawashima Y, Yamamoto H (2019) Ionic liquid-incorporated polymeric nanoparticles as carriers for prevention and at an earlier stage of periodontal disease. Materialia 8. 10.1016/j.mtla.2019.100395

[CR75] Tiwari S, Seijas J, Vazquez-Tato M, Sarkate A, Karnik K, Nikalje A (2017) Facile synthesis of novel coumarin derivatives, antimicrobial analysis, enzyme assay, docking study, ADMET prediction and toxicity study. Molecules 22:1172. 10.3390/molecules22071172 28703783 10.3390/molecules22071172 PMC6152127

[CR76] Tiwari SV, Seijas JA, Vazquez-Tato MP, Sarkate AP, Karnik KS, Nikalje APG (2018) Ionic liquid-promoted synthesis of novel chromone-pyrimidine coupled derivatives, antimicrobial analysis, enzyme assay, docking study and toxicity study. Molecules 23:440. 10.3390/molecules23020440 29462951 10.3390/molecules23020440 PMC6017654

[CR77] ünver Y, Çelik F, Aydın A, Güler HI, Bektaş KI (2024) Synthesis, characterization and biological activity of novel ionic liquids with bis-imidazole moieties: antitumor, antimicrobial effects and molecular docking studies. Org Commun 1314:23–37. 10.25135/acg.oc.163.2401.4014

[CR78] Vereshchagin AN, Frolov NA, Egorova KS, Seitkalieva MM, Ananikov VP (2021) Quaternary ammonium compounds (QACs) and ionic liquids (ILs) as biocides: from simple antiseptics to tunable antimicrobials. Int J Mol Sci 22. 10.3390/ijms22136793 10.3390/ijms22136793 PMC8268321 34202677

[CR79] Vuotto C, Longo F, Balice MP, Donelli G, Varaldo PE (2014) Antibiotic resistance related to biofilm formation in *Klebsiella pneumoniae*. Pathogens 3:743–758. 10.3390/pathogens3030743 25438022 10.3390/pathogens3030743 PMC4243439

[CR80] Wang C, Chen P, Qiao Y, Kang Y, Guo S, Wu D, Wang J, Wu H (2019) Bacteria-activated chlorin e6 ionic liquid based on cation and anion dual-mode antibacterial action for enhanced photodynamic efficacy. Biomater Sci 7:1399–1410. 10.1039/c8bm00990b 30768109 10.1039/c8bm00990b

[CR81] Wang K, Wang J, Li L, Xu L, Feng N, Wang Y, Fei X, Tian J, Li Y (2020) Novel nonreleasing antibacterial hydrogel dressing by a one-pot method. ACS Biomater Sci Eng 6:1259–1268. 10.1021/acsbiomaterials.9b01812 33464855 10.1021/acsbiomaterials.9b01812

[CR82] Wojcieszak M, Kaczmarek DK, Karolak M, Pałkowski Ł, Lewandowska A, Marcinkowska A, Dopierała K, Materna K (2024) Surface-active ionic liquids and surface-active quaternary ammonium salts from synthesis, characterization to antimicrobial properties. Molecules 29. 10.3390/molecules29020443 10.3390/molecules29020443 PMC10819711 38257354

[CR83] Wu X, Tong Z, Li L, Yu H (2013) Toxic effects of imidazolium-based ionic liquids on *Caenorhabditis elegans* : the role of reactive oxygen species. Chemosphere 93:2399–2404. 10.1016/j.chemosphere.2013.08.040 24021415 10.1016/j.chemosphere.2013.08.040

[CR84] Wu X, Shen M, Wang H, He X, Tan J, Wang R, Yang L, Yang H, Qi J, Chen Z, Zhu Q (2023) Evaluation of the efficacy and safety of ionic liquids containing ketoconazole in patients with tinea pedis: a randomized controlled clinical trial. Bioeng Transl Med 8:1–9. 10.1002/btm2.10463 10.1002/btm2.10463 PMC10189433 37206222

[CR85] Xing Y, Hu Y, Zhang X, Zheng D, Ma G, Diao Y, Yue H, Wei W, Zhang S (2025) Cationic alkyl chain length and nanoaggregate form of ionic liquids dominate biocompatibility and toxicity. Nat Commun. 10.1038/s41467-025-62206-x 40721585 10.1038/s41467-025-62206-x PMC12304108

[CR86] Yan C, Nakajima M, Yanagawa M, Tabeta K, Ikeda-Imafuku M, Fukuta T, Hayatsu M, Shibata S, Mitragotri S (2024) Choline and geranate ionic liquid for subgingival biofilm control. Int J Pharm 662:124544. 10.1016/j.ijpharm.2024.124544 39094920 10.1016/j.ijpharm.2024.124544

[CR87] Young GR, Abdelghany TM, Leitch AC, Dunn MP, Blain PG, Lanyon C, Wright MC (2020) Changes in the gut microbiota of mice orally exposed to methylimidazolium ionic liquids. PLoS One 1–22. 10.1371/journal.pone.0229745 10.1371/journal.pone.0229745 PMC7067480 32163446

[CR88] Yu Y, Yang Z, Ren S, Gao Y, Zheng L (2020) Multifunctional hydrogel based on ionic liquid with antibacterial performance. J Mol Liq 299:112185. 10.1016/j.molliq.2019.112185

[CR89] Zhang J, Cheng C, Lu C, Li W, Li B, Wang J, Wang J, Du Z, Zhu L (2022) Comparison of the toxic effects of non-task-specific and task-specific ionic liquids on zebrafish. Chemosphere 294:133643. 10.1016/j.chemosphere.2022.133643 35051520 10.1016/j.chemosphere.2022.133643

[CR90] Zheng Z, Xu Q, Guo J, Qin J, Mao H, Wang B, Yan F (2016) Structure–antibacterial activity relationships of imidazolium-type ionic liquid monomers, poly(ionic liquids) and poly(ionic liquid) membranes: effect of alkyl chain length and cations. ACS Appl Mater Interfaces 8:12684–12692. 10.1021/acsami.6b03391 27145107 10.1021/acsami.6b03391

[CR91] Zheng L, Li J, Yu M, Jia W, Duan S, Cao D, Ding X, Yu B, Zhang X, Xu F-J (2020) Molecular sizes and antibacterial performance relationships of flexible ionic liquid derivatives. J Am Chem Soc 142:20257–20269. 10.1021/jacs.0c10771 33179921 10.1021/jacs.0c10771

[CR92] Zhou C, Zhao C, Dai H-Y, Zhu T-J, Yao J-P, Chen L, Wen J-J, Loh JLC, Zhou X-D, Huang Y (2024) Antibacterial and angiogenic dual-functional fibrous membrane dressing for infected wound healing. Biomed Eng Commun 3:2. 10.53388/bmec2024002

