# Supplementary material for: Insights into the antimicrobial and antibiofilm activities of ionic liquids and their mechanisms of action
Source: Appl Microbiol Biotechnol. 2026 Mar 20;110:112. doi: 10.1007/s00253-026-13728-x (PMC13005822; doi:10.1007/s00253-026-13728-x)
Supplement: Supplementary file 1 [file 253_2026_13728_MOESM1_ESM.docx]

**Insights into the antimicrobial and antibiofilm activities of ionic liquids and their mechanisms of action**

Hadeer M. Bedair^1^, Israa A. Elmasry^1^, Fotouh R. Mansour^2, 3*^

*^1^Department of Microbiology and Immunology, College of Pharmaceutical sciences and drug manufacturing, Misr University for Science and technology, the 6^th^ of October City,* 12566, Egypt*.*

*^2^Pharmaceutical Analytical Chemistry Department, Faculty of Pharmacy, Tanta University, Tanta, 31111, Egypt*

*^3^*Department of Medicinal Chemistry, Faculty of Pharmacy, King Salman International University (KSIU), South Sinai, Egypt

**Full postal address:** Elgeish Street, the medical campus of Tanta University, Tanta, Egypt 31111

**Supplementary Materials**

**Table S1:** Techniques commonly used in ILs characterization and their key applications.

| **Technique Category** | **Specific Technique** | **Measured Parameters / Information** | **Key Applications for ILs** |
| --- | --- | --- | --- |
| **Structural and Chemical Identity** | **Nuclear Magnetic Resonance (NMR)** | Chemical structure, purity, ion pairing, hydrogen bonding, dynamic processes. | **¹H, ¹³C:** Confirm organic cation structure. **¹⁹F, ³¹P:** Identify anions. |
|  | **Fourier-Transform Infrared (FT-IR) Spectroscopy** | Functional groups, molecular vibrations, cation-anion interactions, hydrogen bonding. | Fingerprint identification, detecting impurities (e.g., water, unreacted precursors), studying intermolecular forces. |
|  | **Mass Spectrometry (MS)** | Molecular mass, ion structure, fragmentation pattern, purity. | Confirms cation/anion masses, detects trace impurities and decomposition products. |
|  | **Elemental Analysis (EA)** | Weight percentage of C, H, N, S, O elements. | Verifies synthesis success and final purity by comparing experimental vs. theoretical elemental composition. |
| **Thermal Properties** | **Differential Scanning Calorimetry (DSC)** | Phase transitions: Glass Transition (Tg), Melting (Tm), crystallization, decomposition temperatures. | Determines if an IL is truly liquid at room temp, studies polymorphism, and assesses thermal stability range. |
|  | **Thermogravimetric Analysis (TGA)** | Thermal stability, decomposition temperature, vapor pressure, moisture content. | Measures decomposition onset temperature (Tₐ), evaluates purity (single-step vs. multi-step degradation). |
| **Electrochemical Properties** | **Cyclic Voltammetry (CV) & Electrochemical Impedance Spectroscopy (EIS)** | Electrochemical window, conductivity, diffusion coefficients, redox behavior, viscosity. | **CV:** Determines anodic/cathodic limits for electrochemical applications. **EIS:** Measures ionic conductivity. |
| **Physical and Transport Properties** | **Rheometry** | Viscosity, viscoelastic behavior, shear dependence. | Measures dynamic viscosity (crucial for process design), studies non-Newtonian behavior. |
|  | **Conductivity Meters** | Ionic conductivity. | Direct measurement of conductivity, often correlated with viscosity and temperature. |
|  | **Density Meters / Pycnometry** | Density. | Essential for process design, calculation of molar volume, and thermal expansion coefficients. |
|  | **Surface Tensiometry** | Surface tension. | Important for applications in catalysis, separations, and materials science. |
| **Composition and Purity** | **Karl Fischer Titration (KF)** | Water content. | Small amounts of water CAn alter IL properties |
|  | **Ion Chromatography (IC)** / **High-Performance Liquid Chromatography (HPLC)** | Anion/cation concentration, presence of halide or other ionic impurities. | Quantifies specific ionic impurities (e.g., Cl⁻, Br⁻) from synthesis, checks for ion exchange completeness. |
|  | **UV-Vis Spectroscopy** | Color, presence of chromophoric impurities, metal ion contamination. | Quick check for purity, quantification of certain impurities. |
